# Supplementary material for: Formation of S- and Z-twist supramolecular micro-ropes by peptide stereoisomers
Source: Nat Commun. 2026 Mar 26;17:4424. doi: 10.1038/s41467-026-71043-5 (PMC13183945; doi:10.1038/s41467-026-71043-5)
Supplement: Supplementary file 1 — Supplementary Information [file 41467_2026_71043_MOESM1_ESM.pdf]

Supplementary Information

Formation of S- and Z- twist supramolecular micro-ropes by peptide stereoisomers

Hui Yuan,[1] Zhongyuan Yang,[2] Chengqian Yuan,[3] Sudha Shankar,[1] Aviad Levin,[4] Tiancheng Lv,[5] Zihan Wang,[2] Wei Sun,[5] Jadon Sitton,[6] Pierre-Andre Cazade,[7] Yoav Dan,[8] Yiming Tang,[2] Lihi Adler-Abramovich,[8] Yi Cao,[5] Sigal Rencus-Lazar,[1] Damien Thompson,[7] Dmitry Kurouski,[6] Tuomas P. J. Knowles,[4] Linda J. W. Shimon,[9] Guanghong Wei,[2,*] Bin Xue,[5,*] Rusen Yang,[10,*] and Ehud Gazit[1,*]

Affiliations:

[1]The Shmunis School of Biomedicine and Cancer Research, George S. Wise Faculty of Life Sciences; Tel Aviv University, Tel Aviv 6997801, Israel

[2]Department of Physics, State Key Laboratory of Surface Physics, Key Laboratory for Computational Physical Science (Ministry of Education), Fudan University, Shanghai 200433, People's Republic of China

[3]State Key Laboratory of Biopharmaceutical Preparation and Delivery, Institute of Process Engineering, Chinese Academy of Sciences, Beijing, China

[4]Centre for Misfolding Diseases, Yusuf Hamied Department of Chemistry, University of Cambridge, Lensfield Road, Cambridge CB2 1EW, United Kingdom

[5]National Laboratory of State Microstructure, Department of Physics, Nanjing University, Nanjing, 210093, Jiangsu, China

[6]Department Biochemistry and Biophysics, Texas A&M University, 2128 TAMU, College Station, TX 77843, USA.

[7]Department of Physics, Bernal Institute, University of Limerick, Limerick, V94 T9PX, Ireland

[8]Department of Oral Biology, The Goldschleger School of Dental Medicine, Gray Faculty of Medical and Health Sciences, Tel Aviv University, Tel Aviv 6997801, Israel

[9]Department of Chemical Research Support, Weizmann Institute of Science, Rehovot, 7610001, Israel

[10]Academy of Advanced Interdisciplinary Research, School of Physics, Xidian University, Xi'an 710126, China
*Corresponding author. Email: ehudg@post.tau.ac.il; rsyang@xidian.edu.cn; xuebinnju@nju.edu.cn; ghwei@fudan.edu.cn

This PDF file includes:

Supplementary Notes 1-5

Supplementary Figures 1-92

Supplementary Tables 1-8

Supplementary Video Legends

Other Supplementary Materials for this manuscript include the following:

Supplementary Videos 1-10

Supplementary single crystal data 1-12

Table of Contents

Supplementary Methods 1: Ultra-High-Performance Liquid Chromatography (UHPLC) 5
Supplementary Methods 2: Nuclear magnetic resonance (NMR) 5
Supplementary Methods 3: Scanning electron microscopy (SEM) 5
Supplementary Methods 4: Solubility 5
Supplementary Methods 5: Circular dichroism (CD) spectroscopy 6
Supplementary Figure 1: 7
Supplementary Figure 2: 8
Supplementary Figure 3: 9
Supplementary Figure 4: 10
Supplementary Figure 5: 11
Supplementary Figure 6: 12
Supplementary Figure 7: 13
Supplementary Figure 8: 14
Supplementary Figure 9: 15
Supplementary Figure 10: 15
Supplementary Figure 11: 16
Supplementary Figure 12: 16
Supplementary Figure 13: 17
Supplementary Figure 14: 18
Supplementary Figure 15: 19
Supplementary Figure 16: 19
Supplementary Figure 17: 20
Supplementary Figure 18: 20
Supplementary Figure 19: 21
Supplementary Figure 20: 21
Supplementary Figure 21: 22
Supplementary Figure 22: 22
Supplementary Figure 23: 23
Supplementary Figure 24: 23
Supplementary Figure 25: 24
Supplementary Figure 26: 24

Supplementary Figure 27: 25

Supplementary Figure 28: 26

Supplementary Figure 29: 26

Supplementary Figure 30: 27

Supplementary Figure 31: 28

Supplementary Figure 32: 28

Supplementary Figure 33: 29

Supplementary Figure 34: 30

Supplementary Figure 35: 31

Supplementary Figure 36: 31

Supplementary Figure 37: 32

Supplementary Figure 38: 32

Supplementary Figure 39: 33

Supplementary Figure 40: 34

Supplementary Figure 41: 34

Supplementary Figure 42: 35

Supplementary Figure 43: 36

Supplementary Figure 44: 37

Supplementary Figure 45: 37

Supplementary Figure 46: 38

Supplementary Figure 47: 38

Supplementary Figure 48: 39

Supplementary Figure 49: 39

Supplementary Figure 50: 40

Supplementary Figure 51: 40

Supplementary Figure 52: 41

Supplementary Figure 53: 41

Supplementary Figure 54: 42

Supplementary Figure 55: 42

Supplementary Figure 56: 43

Supplementary Figure 57: 44

Supplementary Figure 58: 45

Supplementary Figure 59: 46
Supplementary Figure 60: 47
Supplementary Figure 61: 48
Supplementary Figure 62: 49
Supplementary Figure 63: 49
Supplementary Figure 64: 50
Supplementary Figure 65: 50
Supplementary Figure 66: 51
Supplementary Figure 67: 51
Supplementary Figure 68: 52
Supplementary Figure 69: 52
Supplementary Figure 70: 53
Supplementary Figure 71: 53
Supplementary Figure 72: 54
Supplementary Figure 73: 54
Supplementary Figure 74: 55
Supplementary Figure 75: 56
Supplementary Figure 76: 57
Supplementary Figure 77: 58
Supplementary Figure 78: 59
Supplementary Figure 79: 59
Supplementary Figure 80: 60
Supplementary Figure 81: 60
Supplementary Figure 82: 61
Supplementary Figure 83: 62
Supplementary Figure 84: 63
Supplementary Figure 85: 64
Supplementary Figure 86: 65
Supplementary Figure 87: 66
Supplementary Figure 88: 67
Supplementary Figure 89: 68
Supplementary Figure 90: 69

Supplementary Figure 91: 70

Supplementary Table 1:.. 71

Supplementary Table 2:.. 72

Supplementary Table 3:.. 73

Supplementary Table 4:.. 74

Supplementary Table 5:.. 75

Supplementary Table 6:.. 76

Supplementary Table 7:.. 77

Supplementary Table 8:.. 78

CheckCIF B-level Alerts and Justification.. 79

Supplementary references: 80

Supplementary Methods 1: Ultra-High-Performance Liquid Chromatography (UHPLC)

The cyclic peptides c-$^{L}W^{L}P$, c-$^{L}W^{D}P$, c-$^{D}W^{L}P$, and c-$^{D}W^{D}P$ were dissolved in CH_3CN: water (1:1) mixture. UHPLC analysis was performed using Dionex/Thermo UltiMate 3000 UHPLC Systems equipped with a diode array UV detector. The analysis employed Waters XSelect Peptide CSH C18 column (5 μm, 4.6 mm x 100 mm) using a 10-minute gradient from 95:5 Water: CH_3CN (both with 0.1% trifluoroacetic acid) to acetonitrile at 254 nm.

Supplementary Methods 2: Nuclear magnetic resonance (NMR)

^{1}H NMR spectra of the cyclic dipeptides c-$^{L}W^{L}P$, c-$^{L}W^{D}P$, c-$^{D}W^{L}P$, c-$^{D}W^{D}P$ and co-crystals of c-$^{L}W^{L}P$/$^{L}W^{D}P$, $^{L}W^{L}P$/c-$^{D}W^{L}P$, c-$^{L}W^{L}P$/c-$^{D}W^{D}P$, c-$^{L}W^{D}P$/c-$^{D}W^{L}P$, c-$^{L}W^{D}P$/c-$^{D}W^{D}P$, and c-$^{D}W^{L}P$/c-$^{D}W^{D}P$ were recorded on a Bruker AV-400 NMR spectrometer using the Topspin software. Chemical shifts are reported in ppm and referenced to the residual solvent peak of $CDCl_3$ (1H, 7.24 ppm).

Supplementary Methods 3: Scanning electron microscopy (SEM)

Samples of 10 μL dipeptide single crystals or co-crystals were drop-casted on clean silicon substrates and then dried under vacuum. The prepared crystals were sputter-coated with an Au film at 16 mA for 45 s using Quorum SC7620 sputter coater to enhance conductivity. SEM images were collected using a JCM-6000PLUS Neo Scope Benchtop scanning electron microscope.

Supplementary Methods 4: Solubility

<u>Making standard curves</u>

Standard curves of peptides were prepared in deionized (DI) water at pH 7.4. To prepare the stock solution, 2 mg of peptide was weighed in a vial and dissolved in 1 mL of DI water. Finally, dilution was made using the same medium to make different concentrations of peptide solutions for the standard curve. The absorbance of the peptide in each medium was scanned using UV-STAR® Microplate, 96 Well, Tecan Spark® series microplate reader.

Saturated solubility determination

The saturated solubility of the peptide in DI water was determined by adding an excess amount of peptide in the water in Eppendorf and shaking it for 2 hours on an orbital shaker at room temperature. The resulting saturated peptide samples were filtered using syringe filters with 0.22 μm pore size. The filtrate was collected and after appropriate dilution with the same solvent, the absorbance of the peptide was taken with a Tecan Spark® series microplate reader at the λ_{max}=240 nm. Standard curves of the peptides were made in duplicates whereas all unknown and saturated samples were loaded in triplicates.

Supplementary Methods 5: Circular dichroism (CD) spectroscopy

Peptides (c-^{L}W^{L}P, c-^{L}W^{D}P, c-^{D}W^{L}P and c-^{D}W^{D}P) were dissolved in DI water at a concentration of 0.5 mg/mL. CD spectra were obtained using a Chirascan V100 CD spectrometer (Applied Photophysics) equipped with quartz cuvettes with an optical path length of 1 mm. Data were collected in 1 nm increments over a wavelength range of 170-400 nm. The baseline spectrum of DI water was also recorded and subtracted from the sample spectra. Data were processed using Pro-Data Viewer software (Applied Photophysics).

Supplementary Figures

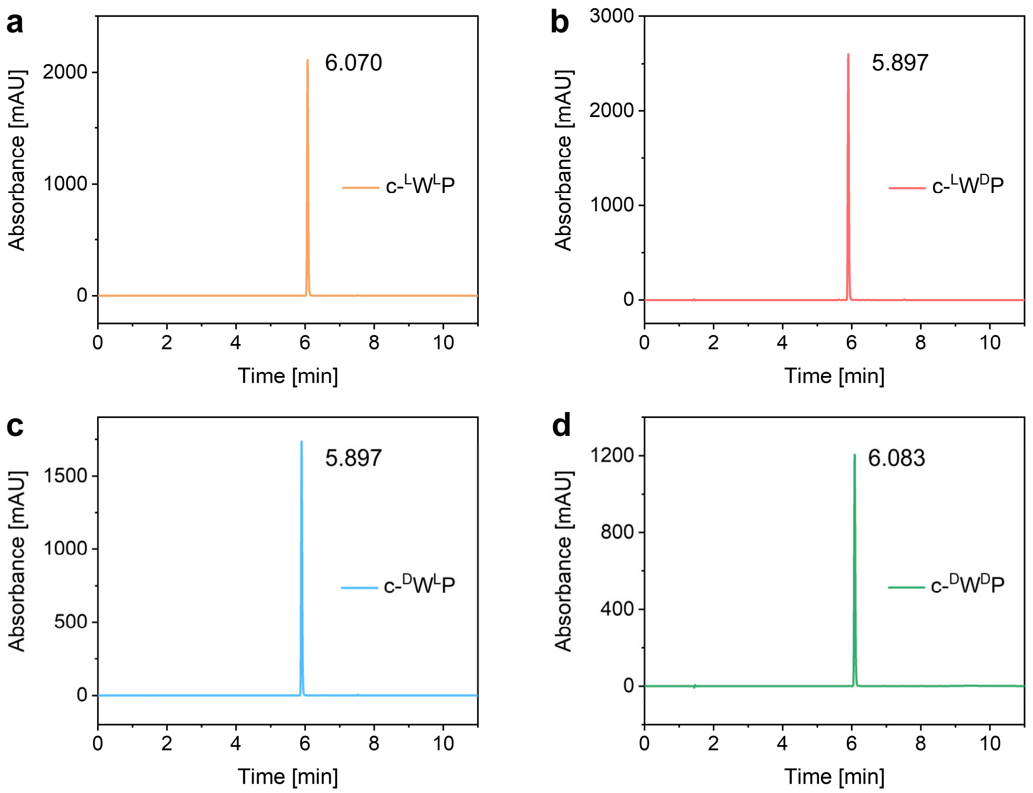

Supplementary Figure 1: HPLC chromatograms of (a) c-$^{L}W^{L}P$, (b) c-$^{L}W^{D}P$, (c) c-$^{D}W^{L}P$, and (d) c-$^{D}W^{D}P$.

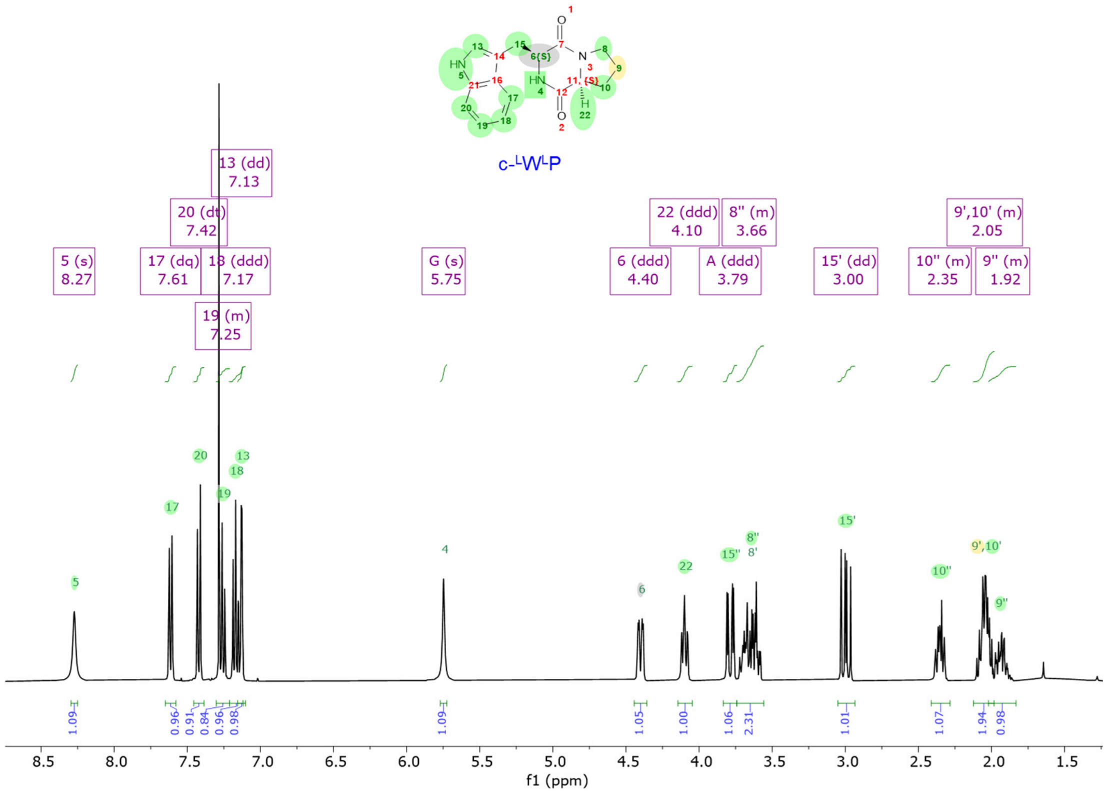

Supplementary Figure 2: ^{1}H NMR spectrum of c-^{L}W^{L}P.

c-^{L}W^{L}P: 1H NMR (400 MHz, $CDCl_3$) δ 8.27 (s, 1H), 7.61 (dq, J = 7.9, 0.9 Hz, 1H), 7.42 (dt, J = 8.2, 0.9 Hz, 1H), 7.30–7.21 (m, 1H), 7.17 (ddd, J = 8.0, 7.1, 1.0 Hz, 1H), 7.13 (dd, J = 2.5, 0.9 Hz, 1H), 5.75 (s, 1H), 4.40 (ddd, J = 11.0, 4.1, 1.6 Hz, 1H), 4.10 (ddd, J = 9.0, 7.0, 1.7 Hz, 1H), 3.79 (ddd, J = 15.1, 3.8, 1.1 Hz, 1H), 3.74–3.56 (m, 2H), 3.00 (dd, J = 15.1, 10.9 Hz, 1H), 2.41–2.28 (m, 1H), 2.12–1.98 (m, 2H), 2.02–1.83 (m, 1H).

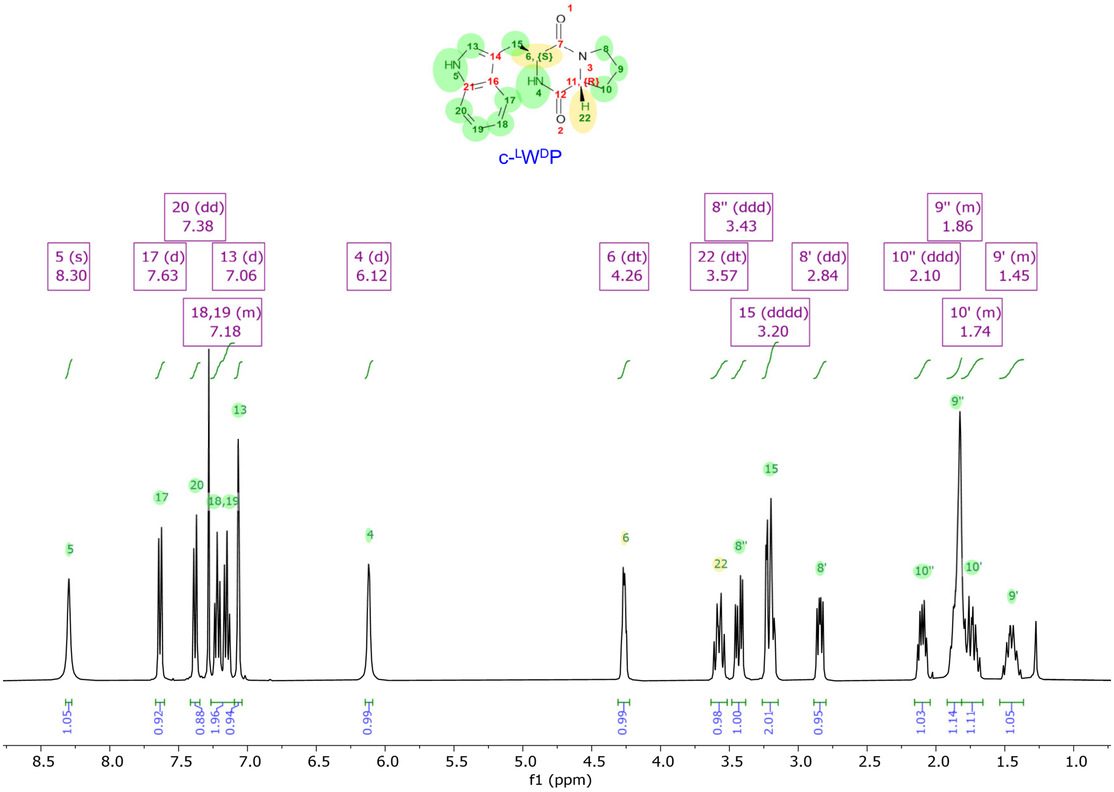

Supplementary Figure 3: ^{1}H NMR spectrum of c-^{L}W^{D}P.

c-^{L}W^{D}P: 1H NMR (400 MHz, $CDCl_3$) δ 8.30 (s, 1H), 7.63 (d, J = 7.8 Hz, 1H), 7.38 (dd, J = 8.1, 1.4 Hz, 1H), 7.27–7.09 (m, 2H), 7.06 (d, J = 2.3 Hz, 1H), 6.12 (d, J = 3.7 Hz, 1H), 4.26 (dt, J = 5.7, 3.9 Hz, 1H), 3.57 (dt, J = 11.6, 8.6 Hz, 1H), 3.43 (ddd, J = 14.6, 6.1, 1.8 Hz, 1H), 3.20 (dddd, J = 11.9, 7.5, 4.4, 2.2 Hz, 2H), 2.84 (dd, J = 10.9, 6.4 Hz, 1H), 2.10 (ddd, J = 12.7, 7.4, 5.7 Hz, 1H), 1.92–1.81 (m, 1H), 1.81–1.66 (m, 1H), 1.54–1.36 (m, 1H).

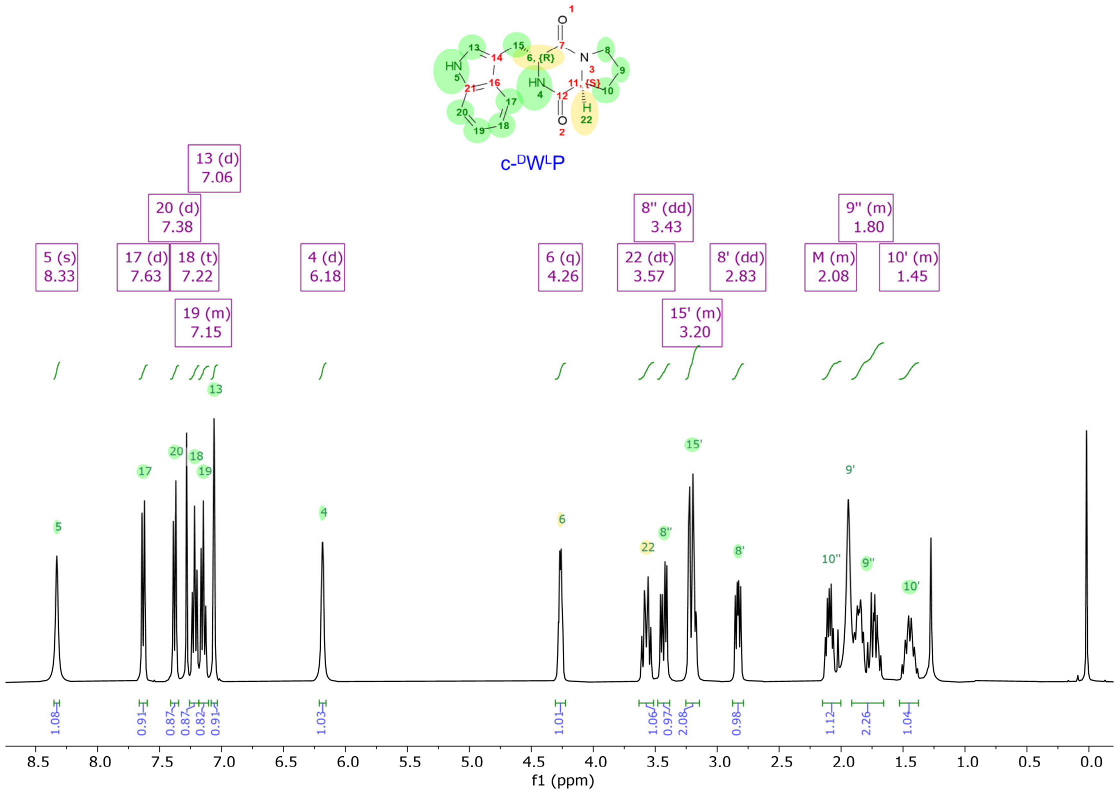

Supplementary Figure 4: ^{1}H NMR spectrum of c-^{D}W^{L}P.

c-^{D}W^{L}P: 1H NMR (400 MHz, $CDCl_3$) δ 8.33 (s, 1H), 7.63 (d, J = 7.9 Hz, 1H), 7.38 (d, J = 8.1 Hz, 1H), 7.22 (t, J = 7.5 Hz, 1H), 7.18–7.10 (m, 1H), 7.06 (d, J = 2.3 Hz, 1H), 6.18 (d, J = 3.7 Hz, 1H), 4.26 (q, J = 4.7 Hz, 1H), 3.57 (dt, J = 12.0, 8.5 Hz, 1H), 3.43 (dd, J = 14.6, 6.0 Hz, 1H), 3.25–3.14 (m, 2H), 2.83 (dd, J = 10.9, 6.3 Hz, 1H), 2.15–2.00 (m, 1H), 1.91–1.66 (m, 2H), 1.53–1.38 (m, 1H).

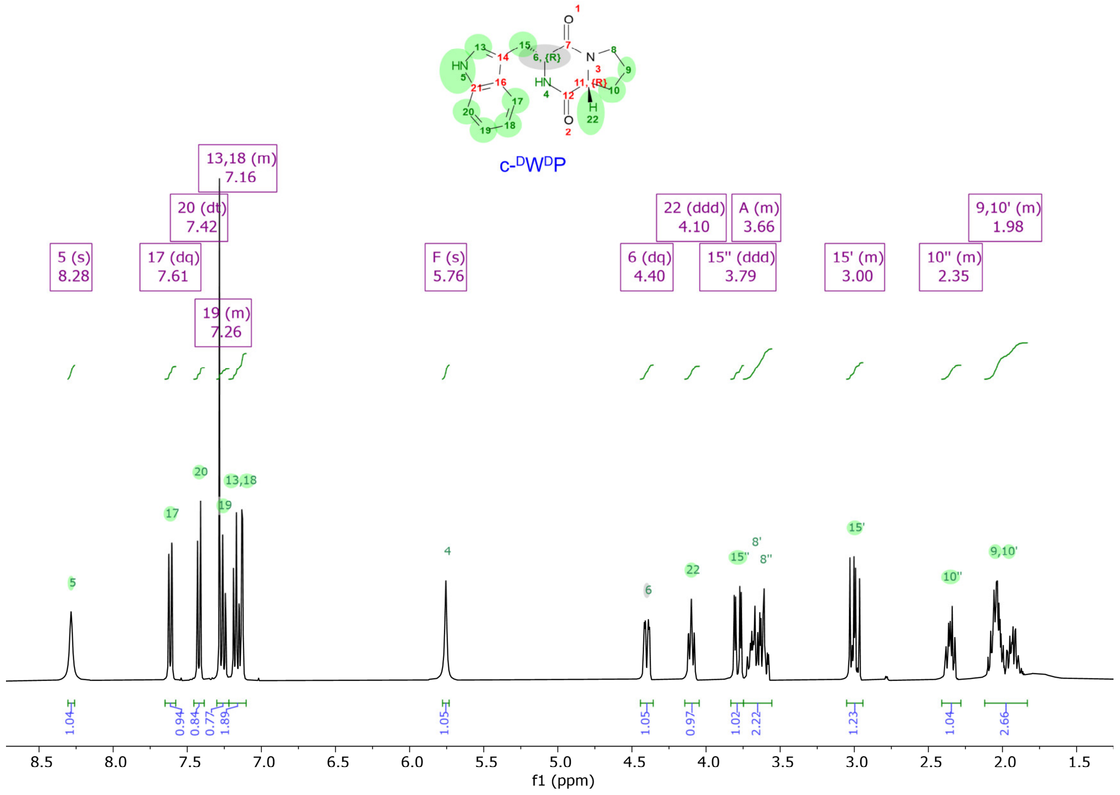

Supplementary Figure 5: ^{1}H NMR spectrum of c-^{D}W^{D}P.

c-^{D}W^{D}P: 1H NMR (400 MHz, $CDCl_3$) δ 8.28 (s, 1H), 7.61 (dq, J = 7.9, 0.9 Hz, 1H), 7.42 (dt, J = 8.2, 0.9 Hz, 1H), 7.30–7.22 (m, 1H), 7.22–7.10 (m, 2H), 5.76 (s, 1H), 4.40 (dq, J = 10.8, 1.5 Hz, 1H), 4.10 (ddd, J = 9.0, 6.9, 1.7 Hz, 1H), 3.79 (ddd, J = 15.1, 3.8, 1.1 Hz, 1H), 3.75–3.56 (m, 2H), 3.05–2.94 (m, 1H), 2.41–2.28 (m, 1H), 2.12–1.83 (m, 3H).

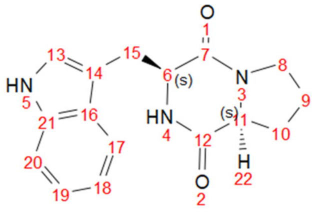

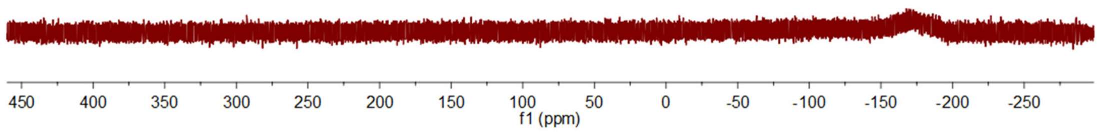

Supplementary Figure 6: ^{19}F NMR (500 MHz) spectrum of c-^{L}W^{L}P (powder form) in DMSO-d6 with 1%(v/v) TMS (Tetramethylsilane).

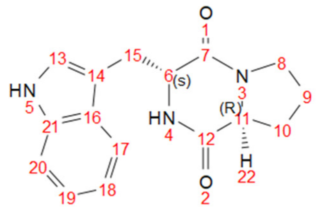

c-^{L}W^{D}P

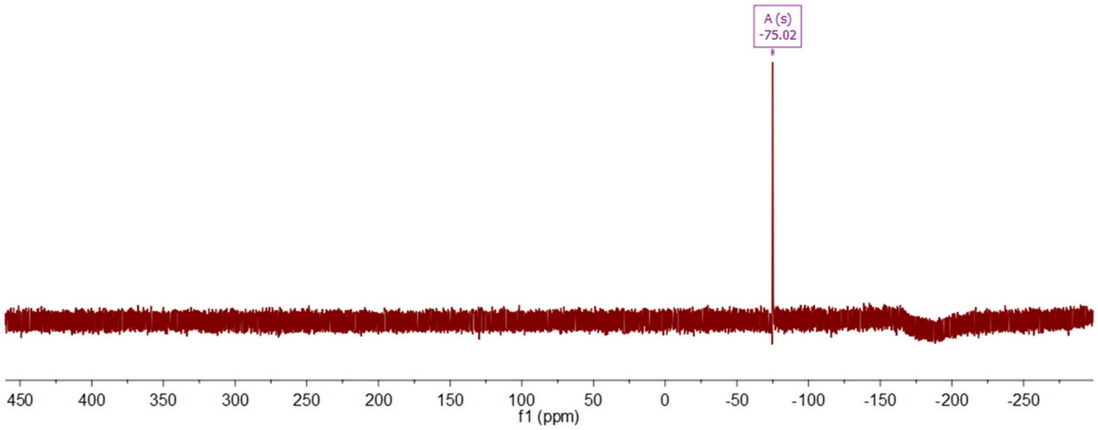

Supplementary Figure 7: ^{19}F NMR (500 MHz) spectrum of c-^{L}W^{D}P (powder form) in DMSO-d6 with 1%(v/v) TMS (Tetramethylsilane).

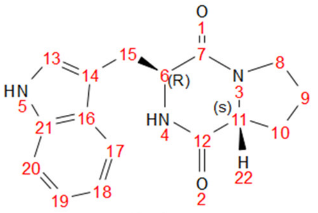

c-^{D}W^{L}P

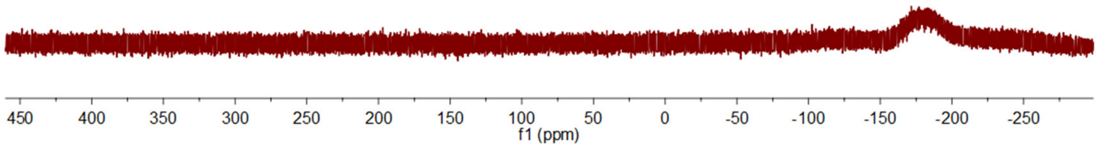

Supplementary Figure 8: ^{19}F NMR (500 MHz) spectrum of c-^{D}W^{L}P (powder form) in DMSO-d6 with 1%(v/v) TMS (Tetramethylsilane).

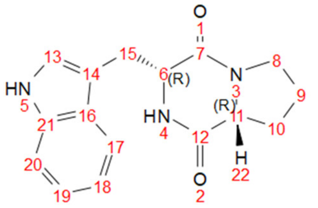

c-^{D}W^{D}P

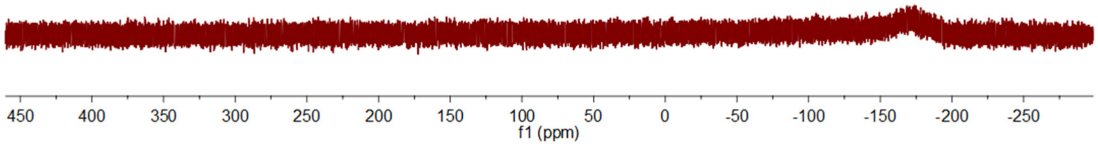

Supplementary Figure 9: ^{19}F NMR (500 MHz) spectrum of c-^{D}W^{D}P (powder form) in DMSO-d6 with 1%(v/v) TMS (Tetramethylsilane).

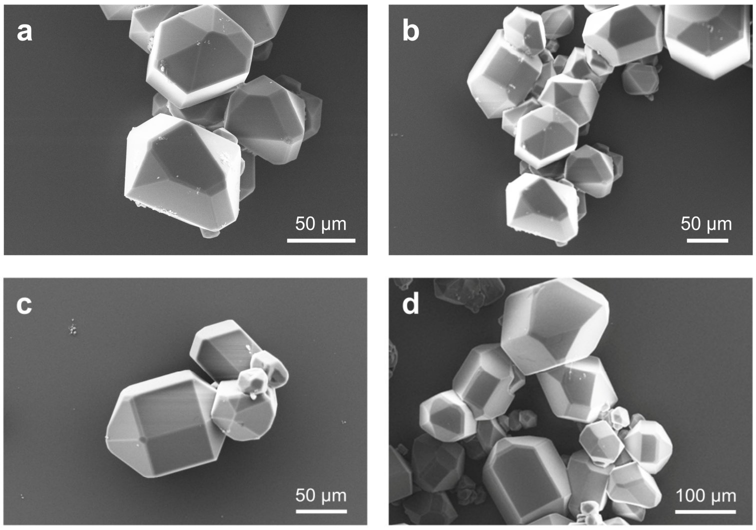

Supplementary Figure 10: (a-d) SEM images of c-^{L}W^{L}P assemblies.

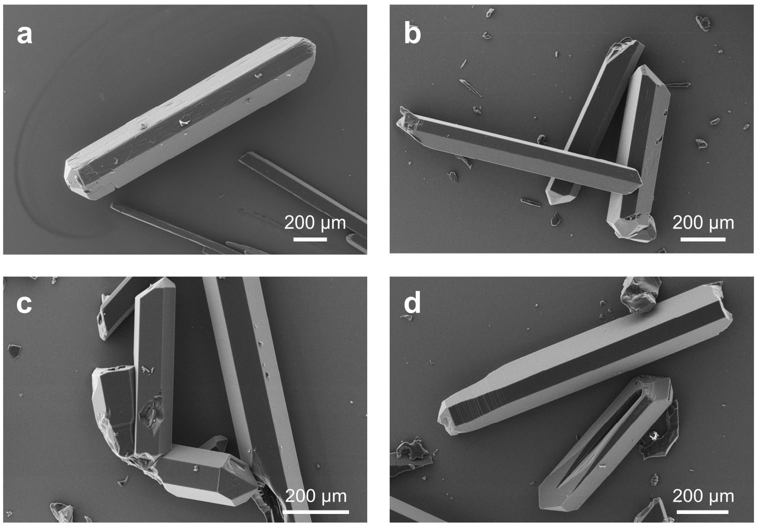

Supplementary Figure 11: (a-d) SEM images of c-$^{L}W^{D}P$ assemblies.

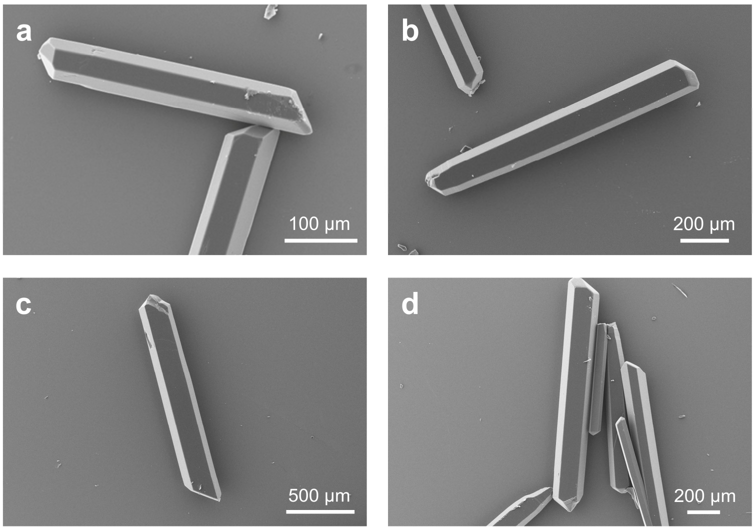

Supplementary Figure 12: (a-d) SEM images of c-$^{D}W^{L}P$ assemblies.

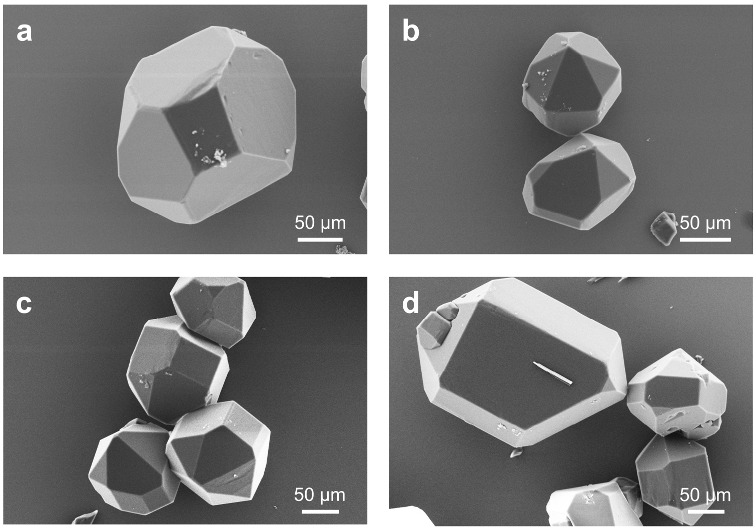

Supplementary Figure 13: (a-d) SEM images of c-^{D}W^{D}P assemblies.

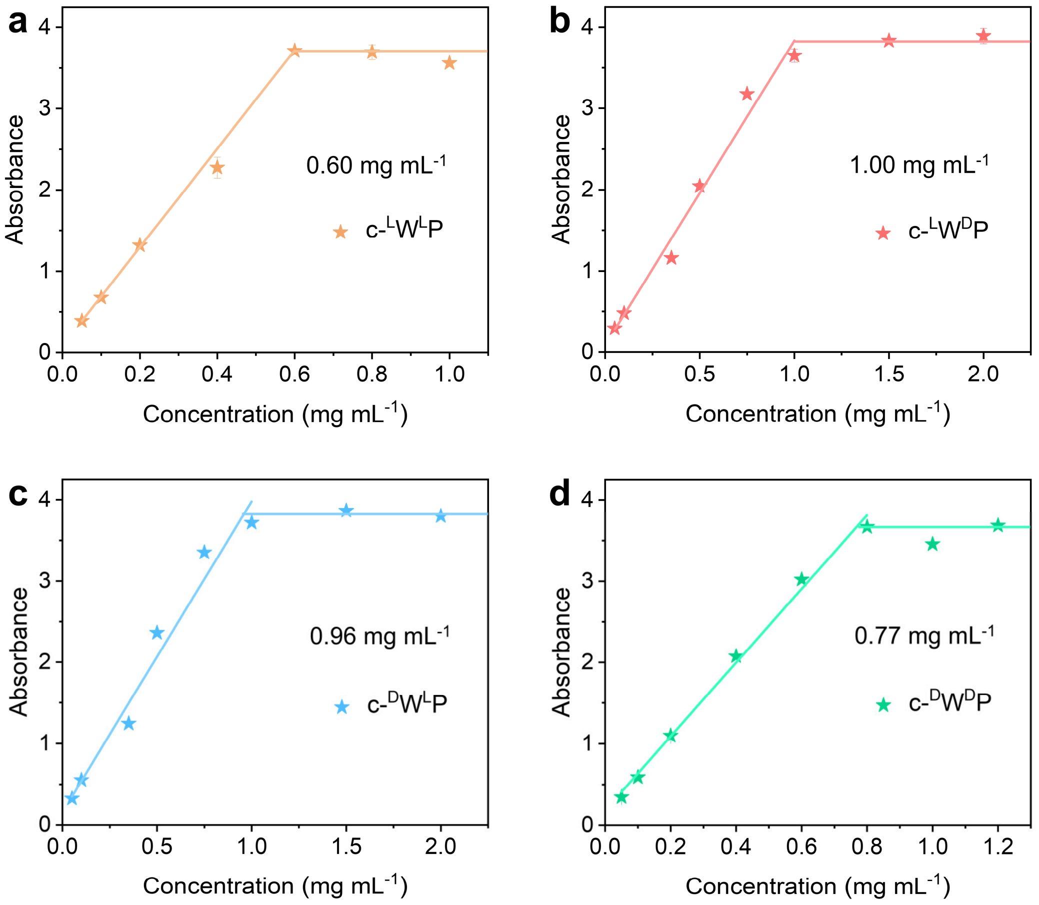

Supplementary Figure 14: Solubility of (a) c-^{L}W^{L}P, (b) c-^{L}W^{D}P, (c) c-^{D}W^{L}P, and (d) c-^{D}W^{D}P.

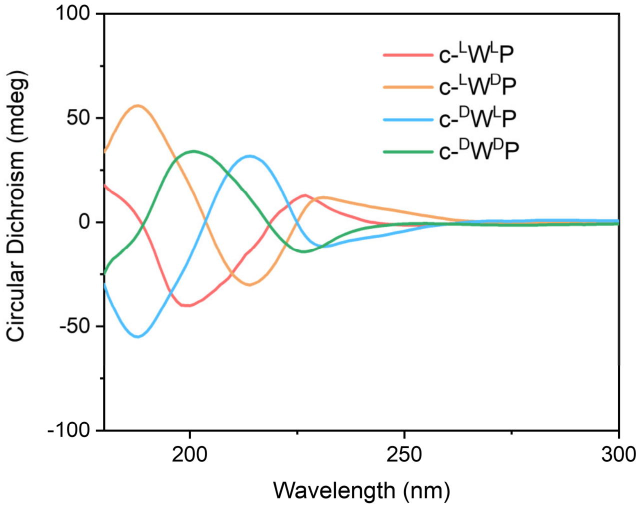

Supplementary Figure 15: CD spectra of c-^{L}W^{L}P, c-^{L}W^{D}P, c-^{D}W^{L}P, and c-^{D}W^{D}P molecules.

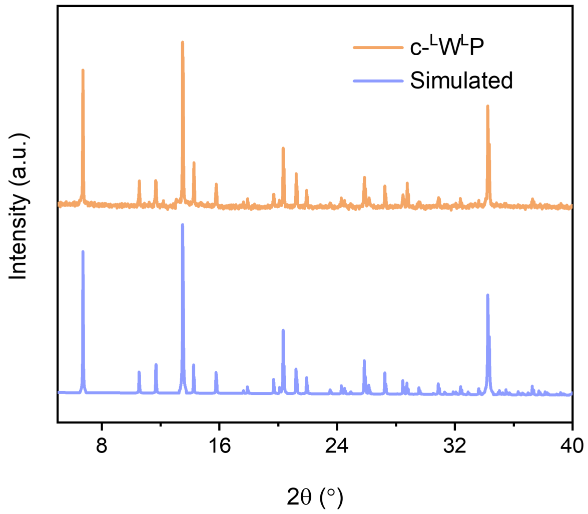

Supplementary Figure 16: Powder X-ray diffraction pattern of c-^{L}W^{L}P assemblies.

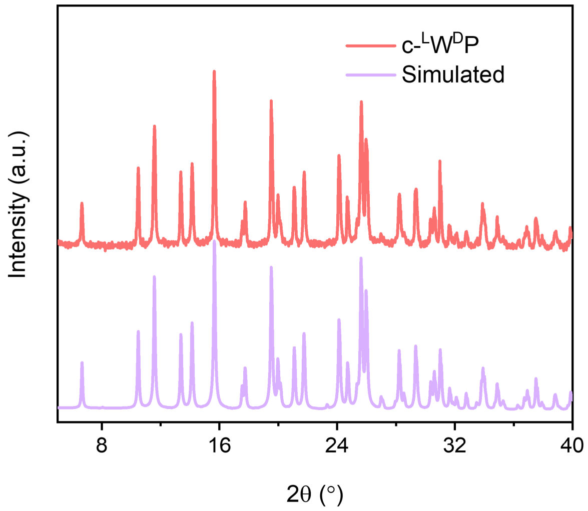

Supplementary Figure 17: Powder X-ray diffraction pattern of c-^{L}W^{D}P assemblies.

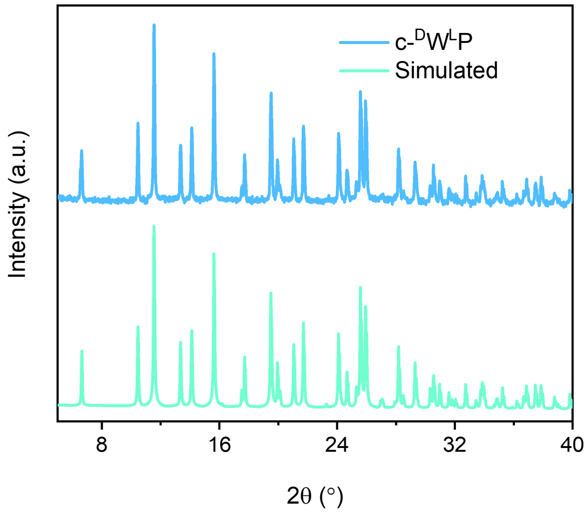

Supplementary Figure 18: Powder X-ray diffraction pattern of c-^{D}W^{L}P assemblies.

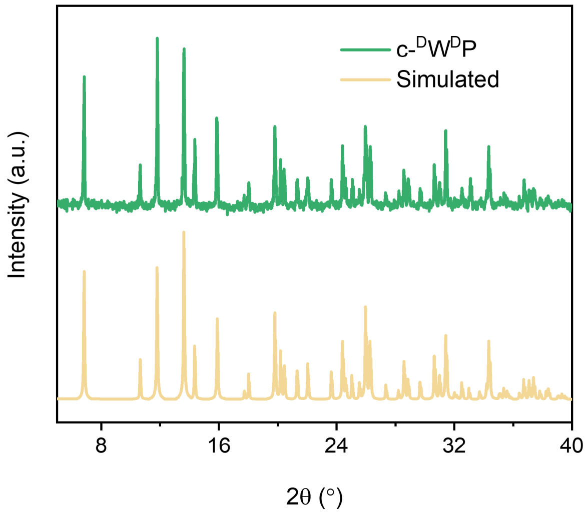

Supplementary Figure 19: Powder X-ray diffraction pattern of c-^{D}W^{D}P assemblies.

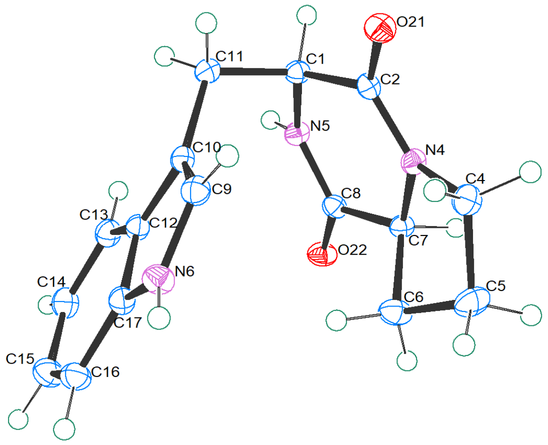

Supplementary Figure 20: ORTEP diagram of the c-^{L}W^{L}P crystal with ellipsoid probability of 50%. CCDC ref. no. 2465098.

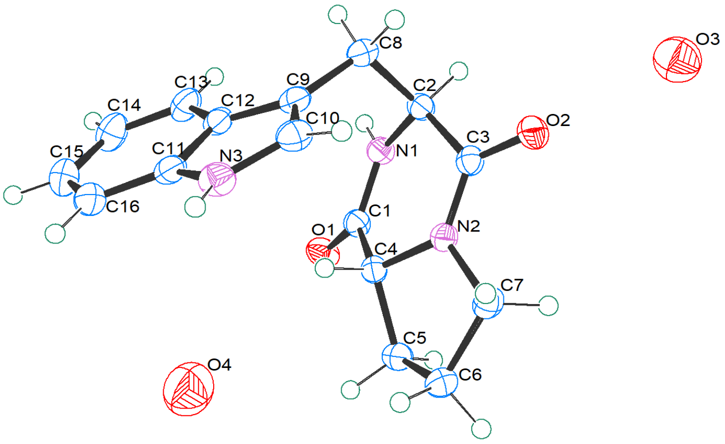

Supplementary Figure 21: ORTEP diagram of the c-^{L}W^{D}P crystal with ellipsoid probability of 50%. CCDC ref. no. 2465099.

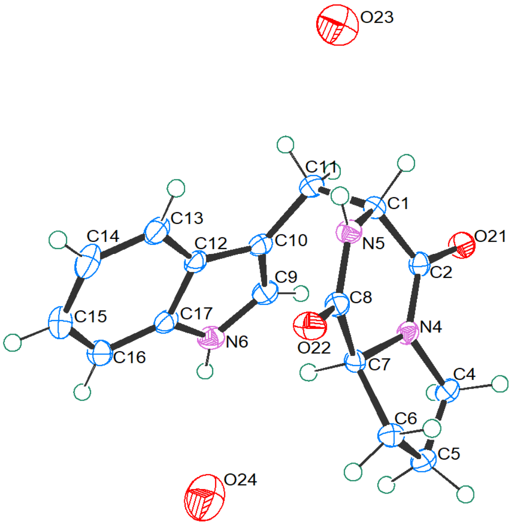

Supplementary Figure 22: ORTEP diagram of the c-^{D}W^{L}P crystal with ellipsoid probability of 50%. CCDC ref. no. 2465100.

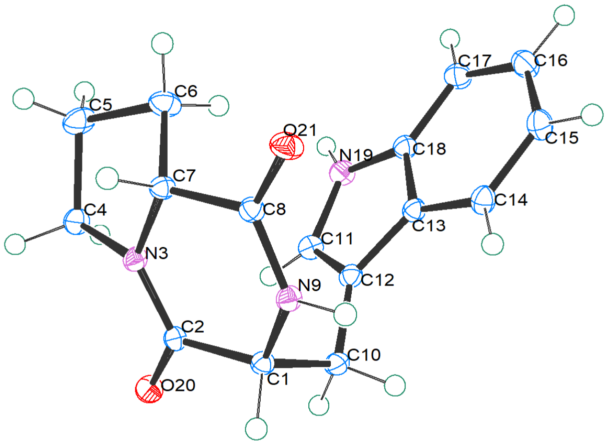

Supplementary Figure 23: ORTEP diagram of the c-^{D}W^{D}P crystal with ellipsoid probability of 50%. CCDC ref. no. 2465101.

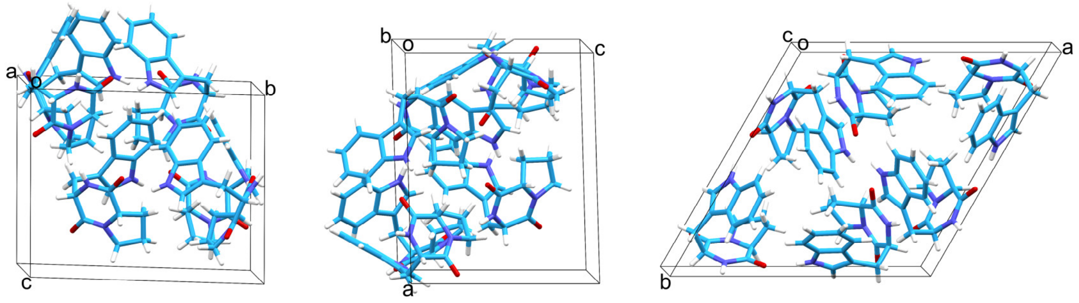

Supplementary Figure 24: Unit cell of c-^{L}W^{L}P assemblies visualized along different directions.

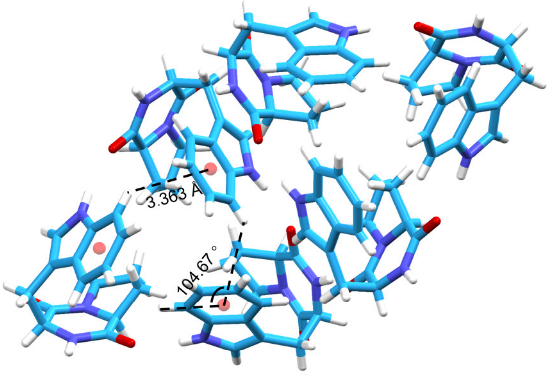

Supplementary Figure 25: Edge-to-face interactions stabilizing the adjacent helices in c-^{L}W^{L}P assemblies.

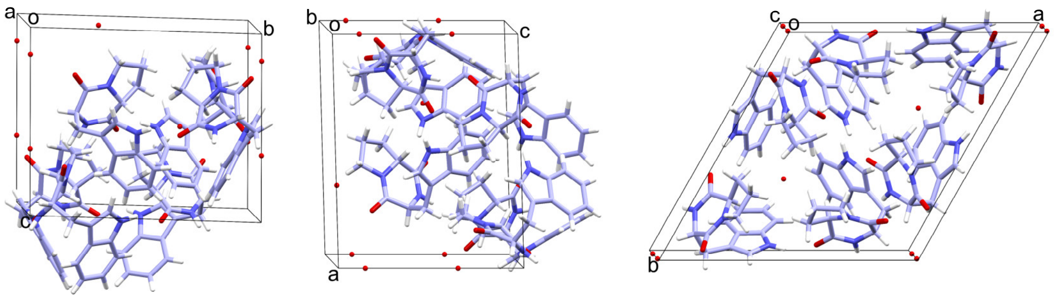

Supplementary Figure 26: Unit cell of c-^{L}W^{D}P assemblies visualized along different directions.

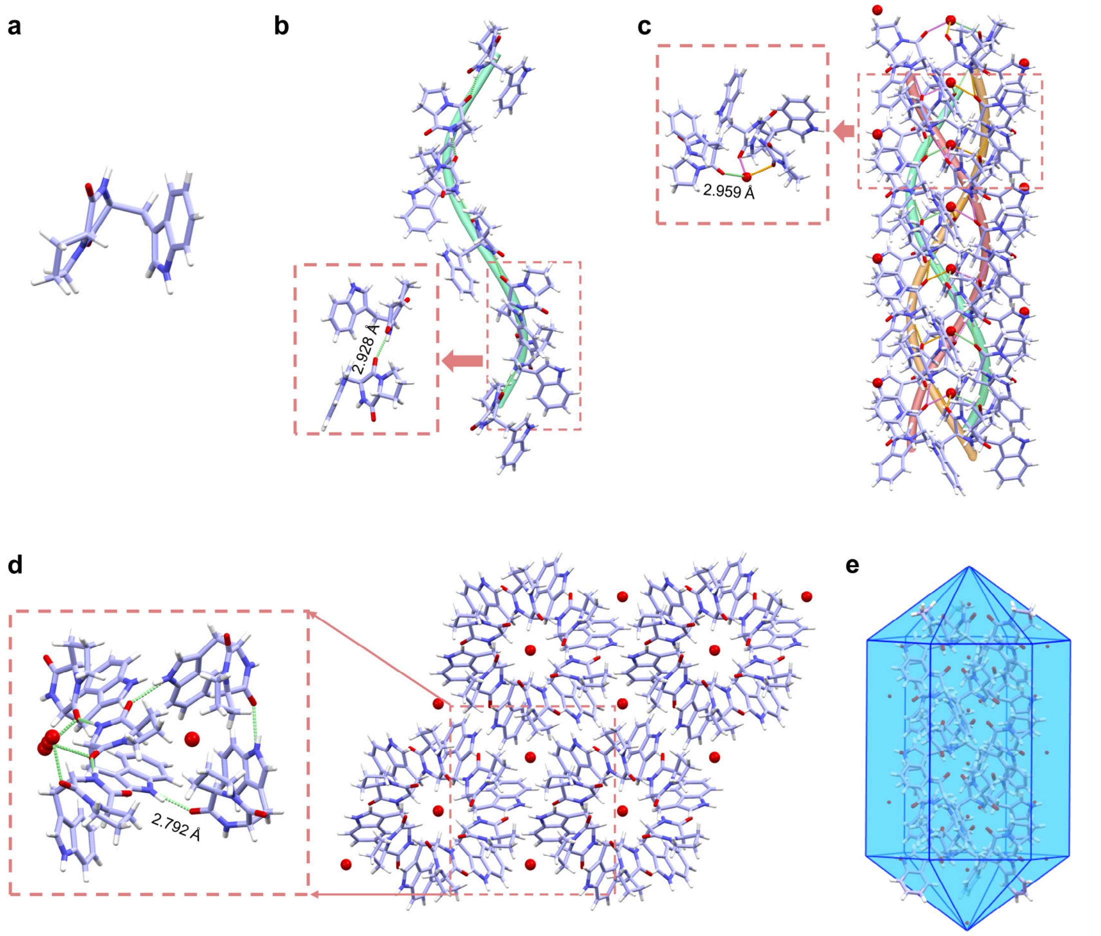

Supplementary Figure 27: Supramolecular packing of c-^{L}W^{D}P assemblies. (a) Asymmetric unit. (b) Supramolecular packing into a single-helical strand. (c) Three helical strands twisting together into a S-supramolecular triple-helical structure. (d) H-bonds connecting the adjacent triple-helical conformations. (e) Hexagonal prism-like morphologies predicted by the BFDH method.

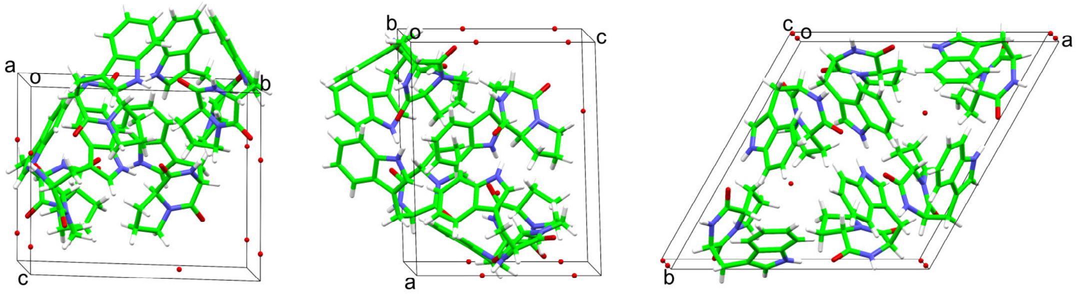

Supplementary Figure 28: Unit cell of c-$^{D}W^{L}P$ assemblies visualized along different directions.

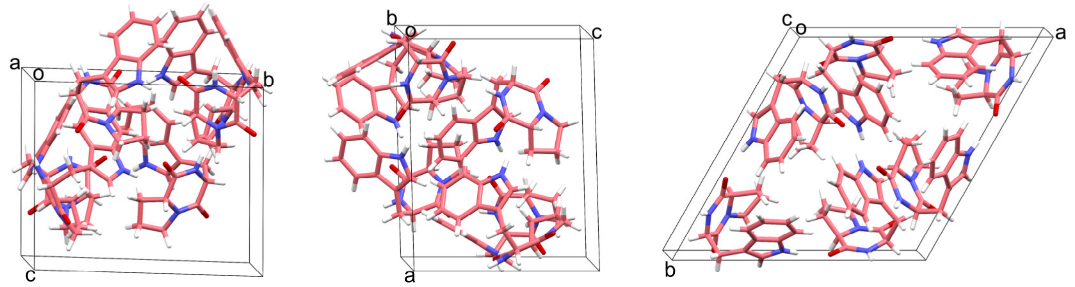

Supplementary Figure 29: Unit cell of c-$^{D}W^{D}P$ assemblies visualized along different directions.

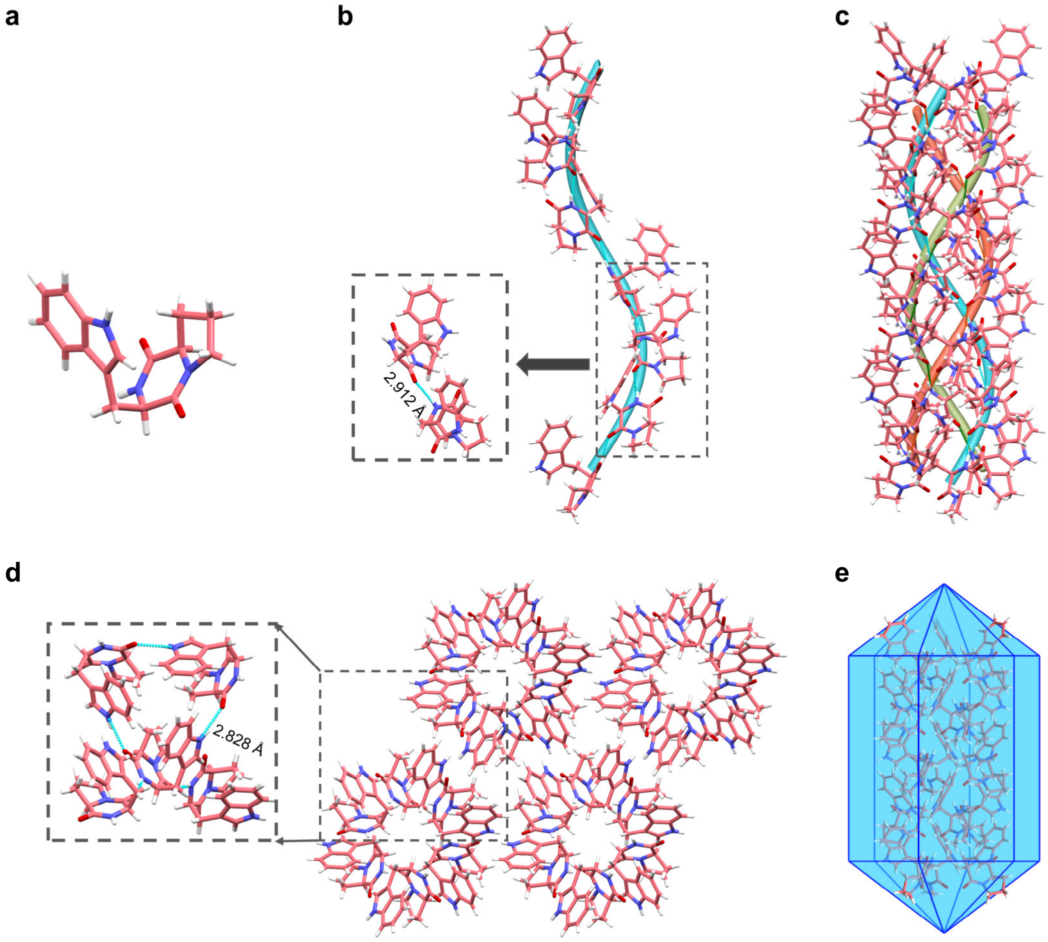

Supplementary Figure 30: Supramolecular packing of c-^{D}W^{D}P assemblies. (a) Asymmetric unit. (b) Supramolecular packing into a single-helical strand. (c) Three helical strands twisting together into a Z-supramolecular triple-helical structure. (d) H-bonds connecting the adjacent triple-helical conformations. (e) Hexagonal prism-like morphologies predicted by the BFDH method.

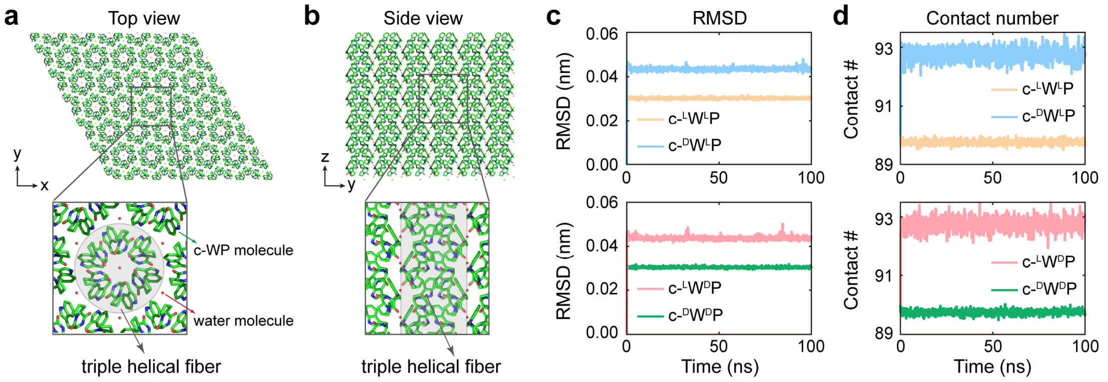

Supplementary Figure 31: The packing and structural stability of the four distinct c-WP crystals. (a, b) Two different views of a 6 × 6 × 6 supercell crystal structure representing the initial conformation of MD simulations: (a) top view and (b) side view. The detailed molecular packings in the crystal structure are highlighted by enlarged views. (c-d) Time evolution of (c) all-atom RMSD of c-WP molecules and (d) inter-molecular contact number during the simulation of the four crystals. The RMSD and the inter-molecular contact numbers of each system remain stable over the 100 ns simulation time, confirming the stability of the constructed crystal structures.

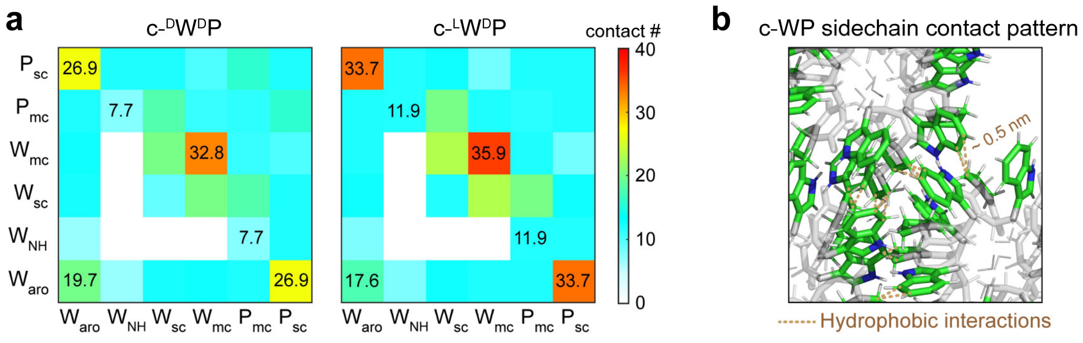

Supplementary Figure 32: (a) Intermolecular contact probability maps between different groups within each c-WP molecule in a single unit cell of the c-$^{D}W^{D}P$ (left) and c-$^{L}W^{D}P$ (right) crystals. (b) A representative snapshot of the c-WP sidechain contacts between different helical bundles in the triple-helical crystal structures.

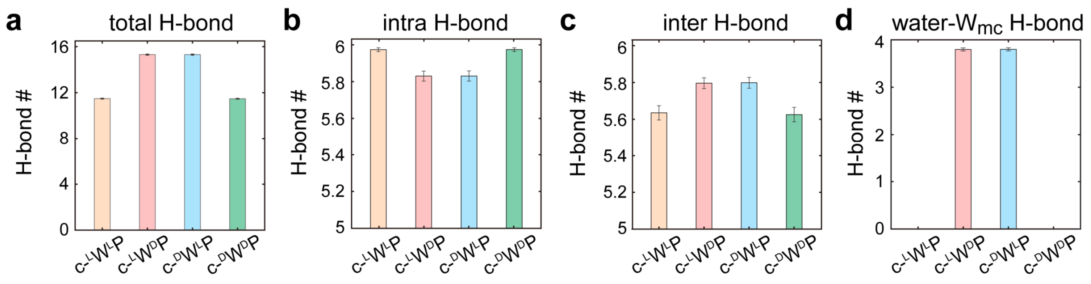

Supplementary Figure 33: Comparison of different H-bond modes across the four c-WP crystals. (a) Number of total intermolecular H-bonds per unit cell. (b) Number of intra-helical H-bonds, corresponding specifically to W_{mc}–W_{mc} H-bonds. (c) Number of inter-helical H-bonds, defined as W_{NH}–P_{mc} H-bonds. (d) Number of H-bonds formed between water molecules and W_{mc} segments (water–W_{mc} H-bonds). H-bonding patterns were categorized into three main types: intra-helical H-bonds between Trp main chains (W_{mc}–W_{mc}), inter-helical H-bonds between the Trp NH group and the Pro main chain (W_{NH}–P_{mc}), and H-bonds between structural water molecules and Trp main chains (water-W_{mc}). Error bars represent the standard deviation of the corresponding values over the last 50 ns of the simulations.

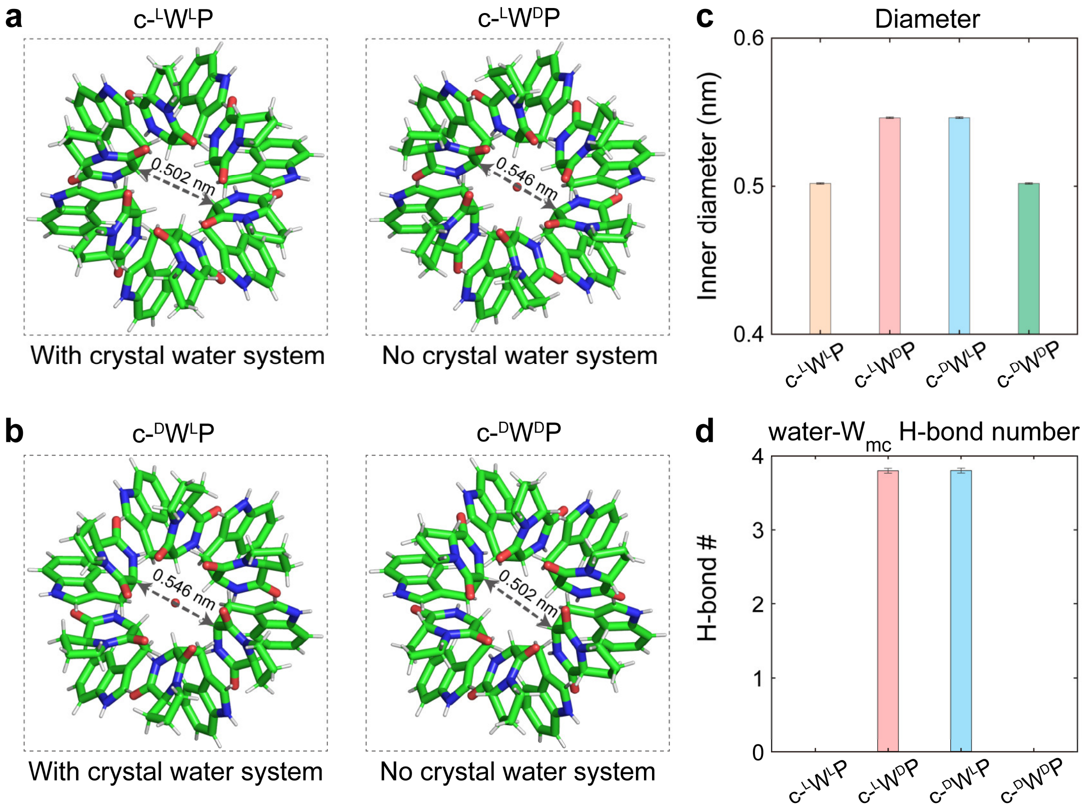

Supplementary Figure 34: Comparison of the inner diameters of triple-helical structures in the four different c-WP crystals. (a,b) Cross-section views of the triple-helical structures in (a) c-^{L}W^{L}P (left) and c-^{L}W^{D}P (right), and (b) c-^{D}W^{L}P (left) and c-^{D}W^{D}P (right) crystals. The inner diameter is indicated with black dashed lines. (c) Comparison of the inner diameters among the four crystals. Hydrated crystals (c-^{D}W^{L}P and c-^{L}W^{D}P) possess larger diameter than dehydrated ones (c-^{L}W^{L}P and c-^{D}W^{D}P), suggesting their capacity to accommodate more water molecules. This structural feature contributes to the enhanced H-bond interactions observed in hydrated crystals. (d) Water-W$_{mc}$ H-bonds in the four crystals. Error bars represent the standard deviation of the corresponding values over the last 50 ns of the simulations.

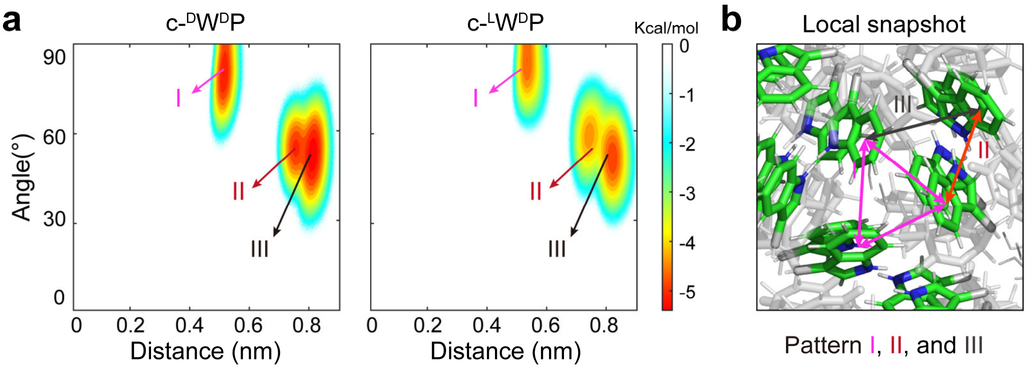

Supplementary Figure 35: Aromatic interactions in the c-^{D}W^{D}P and c-^{L}W^{D}P crystals. (a) Free energy landscapes of π–π angle versus distance for c-^{D}W^{D}P (right) and c-^{L}W^{D}P (left) systems. Pattern I corresponds to the most favorable stacking configuration, whereas Patterns II and III correspond to unfavorable stacking configurations. (b) Local structural snapshot illustrating the spatial arrangement of the dominant π–π stacking mode (Pattern I) alongside the weak stacking modes (Patterns II and III) within the crystal structure.

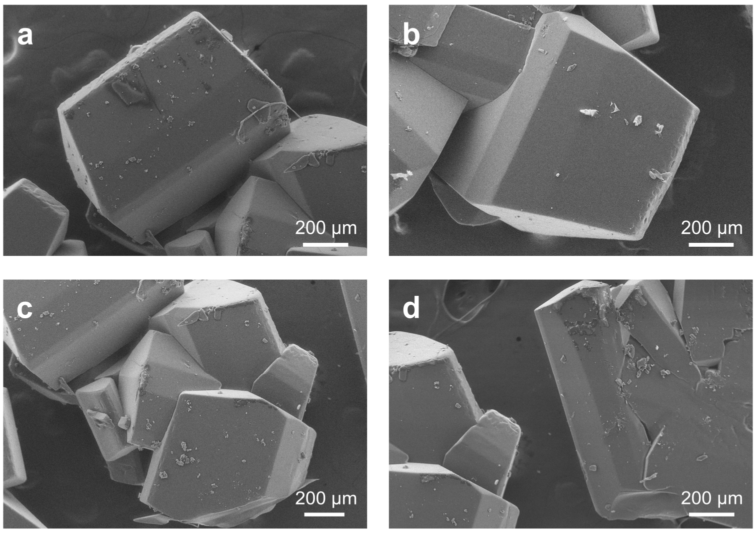

Supplementary Figure 36: SEM images of O-P assemblies.

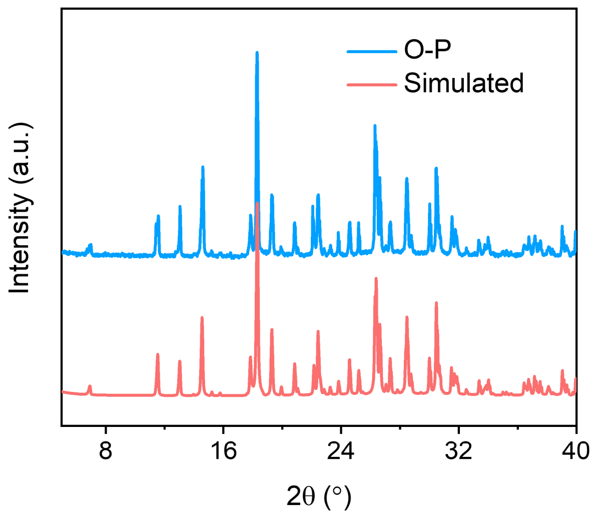

Supplementary Figure 37: XRD pattern of O-P assemblies.

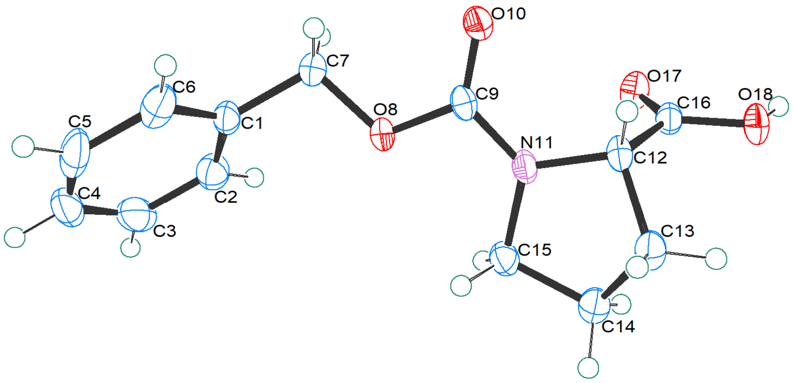

Supplementary Figure 38: ORTEP diagram of the O-P crystal with ellipsoid probability of 50%. CCDC ref. no. 2465132.

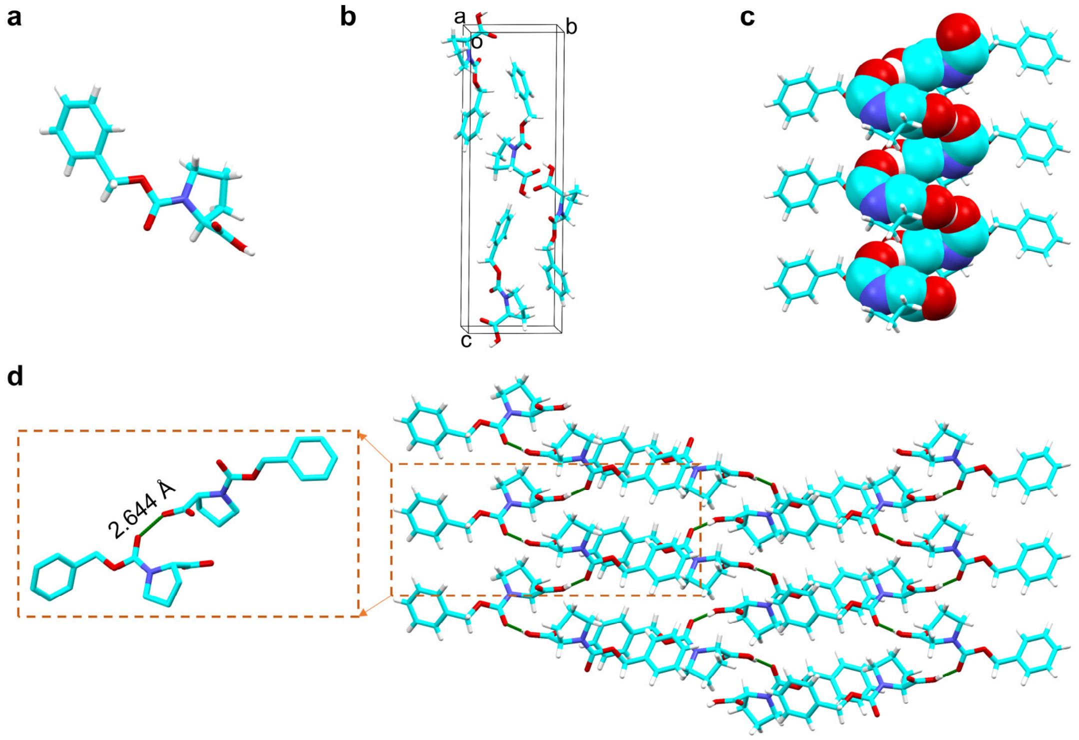

Supplementary Figure 39: The molecular arrangement of O-P showing a supramolecular single-helical structure. (a-d) Self-assembly of O-P. (a) Asymmetric unit. (b) Unit cell. (c) The supraocular stacking into a single helix. (d) Packing of the single helix into a higher-order arrangement. Color code: light blue, C; periwinkle blue, N; Red, O; White, H.

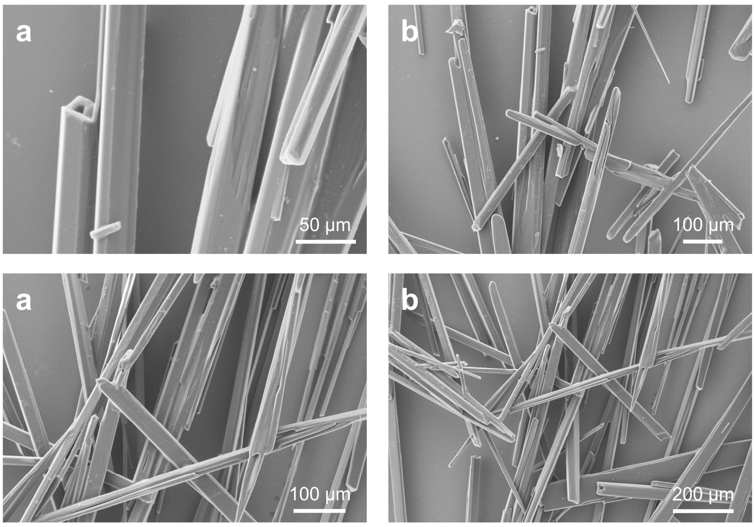

Supplementary Figure 40: (a-d) SEM images of c-VP assemblies.

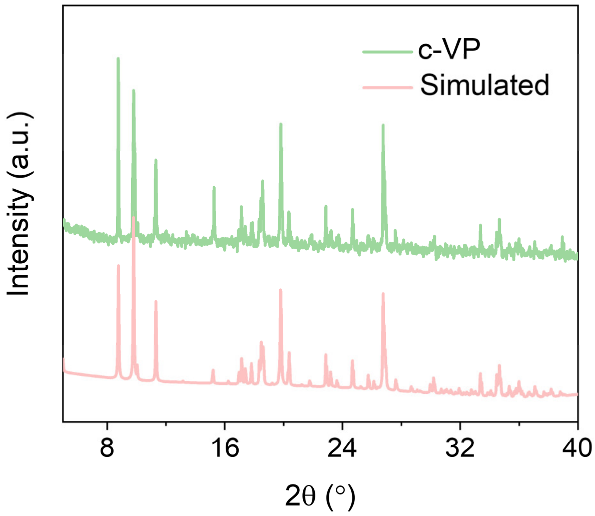

Supplementary Figure 41: XRD pattern of c-VP assemblies.

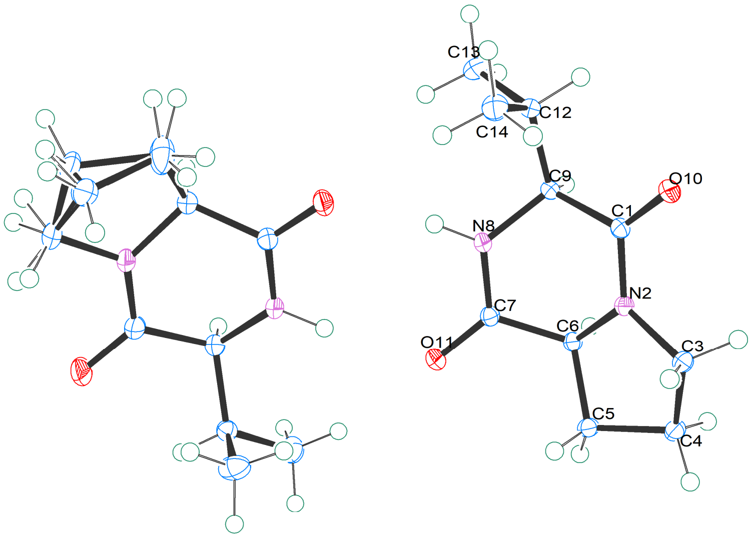

Supplementary Figure 42: ORTEP diagram of the c-VP crystal with ellipsoid probability of 50%. CCDC ref. no. 2504998.

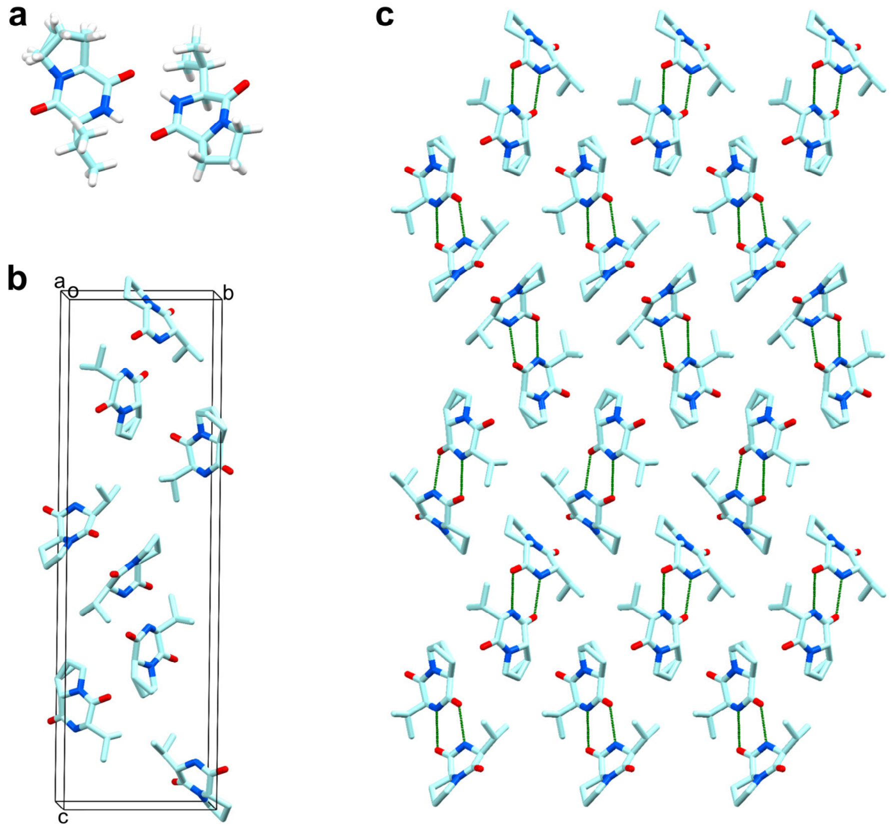

Supplementary Figure 43: The molecular arrangement of c-VP showing a non-helical structure. (a-c) Self-assembly of c-VP. (a) Asymmetric unit. (b) Unit cell. (c) The dimer structure formed by supramolecular stacking. Color code: greyish olive green, C; periwinkle blue, N; Red, O; White, H.

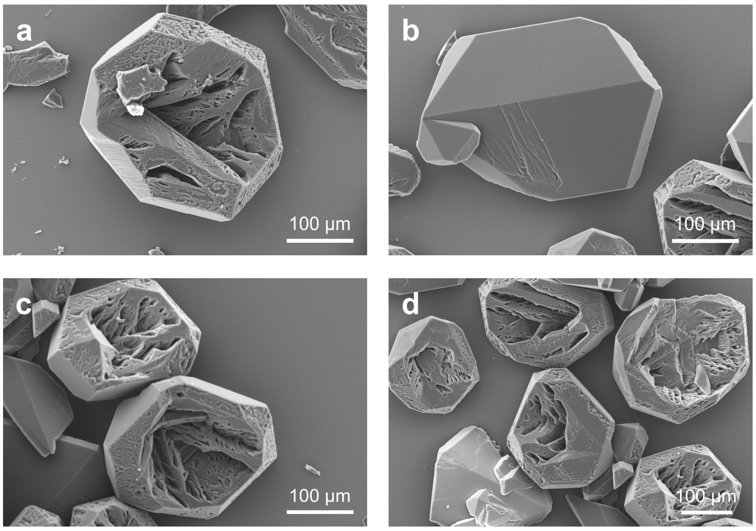

Supplementary Figure 44: (a-d) SEM images of c-^{L}W^{L}P/c-^{L}W^{D}P co-assemblies.

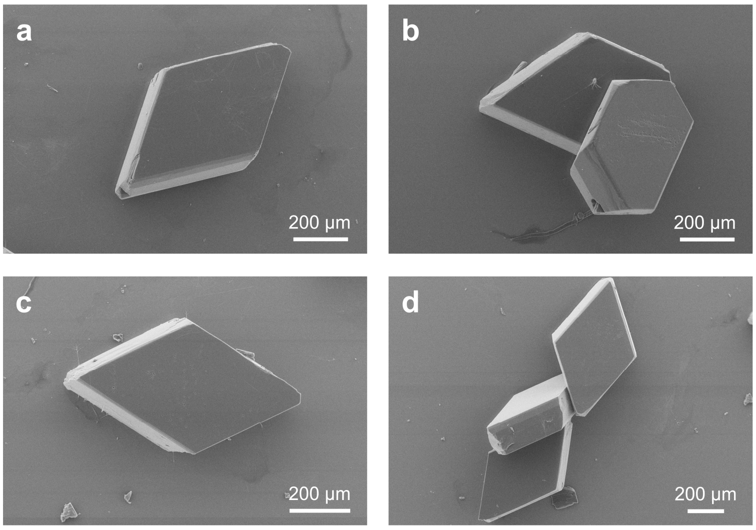

Supplementary Figure 45: (a-d) SEM images of c-^{L}W^{L}P/c-^{D}W^{L}P co-assemblies.

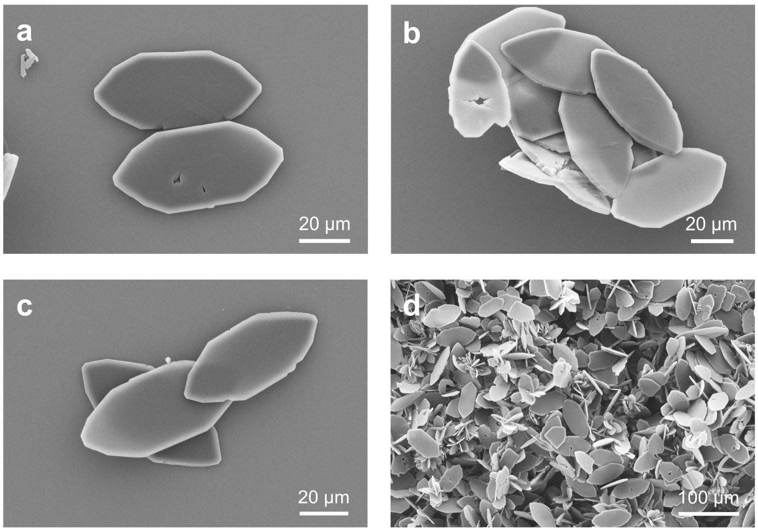

Supplementary Figure 46: (a-d) SEM images of c-$^{L}W^{L}P$/c-$^{D}W^{D}P$ co-assemblies.

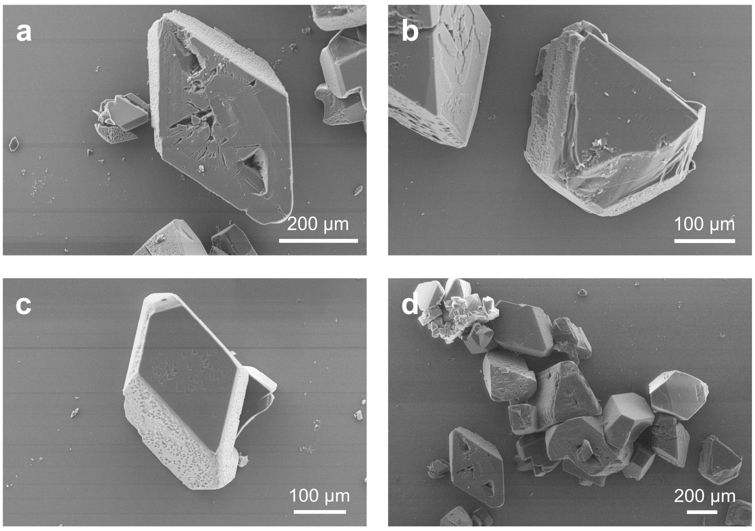

Supplementary Figure 47: (a-d) SEM images of c-$^{L}W^{D}P$/c-$^{D}W^{L}P$ co-assemblies.

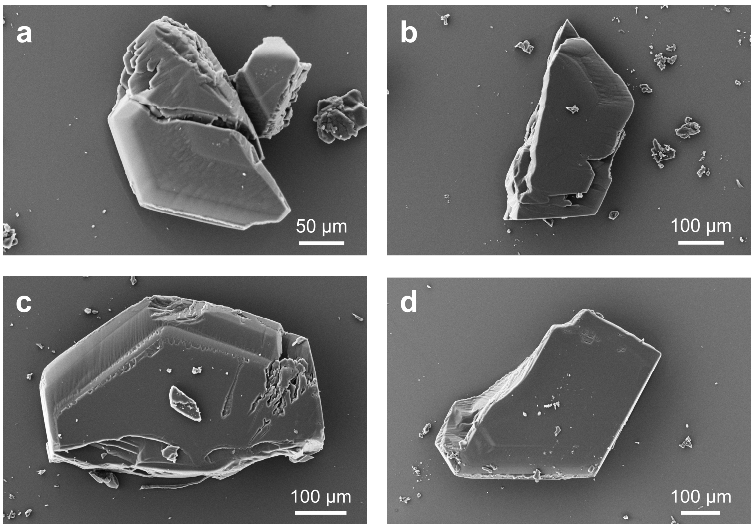

Supplementary Figure 48: (a-d) SEM images of c-^{L}W^{D}P/c-^{D}W^{D}P co-assemblies.

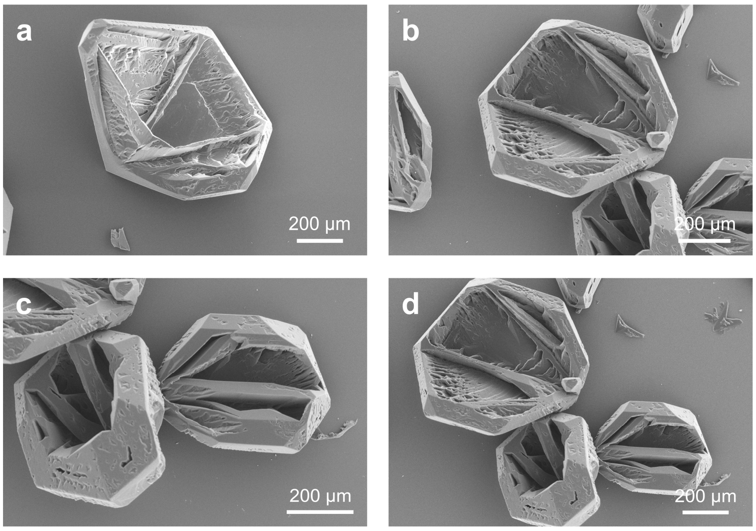

Supplementary Figure 49: (a-d) SEM images of c-^{D}W^{L}P/c-^{D}W^{D}P co-assemblies.

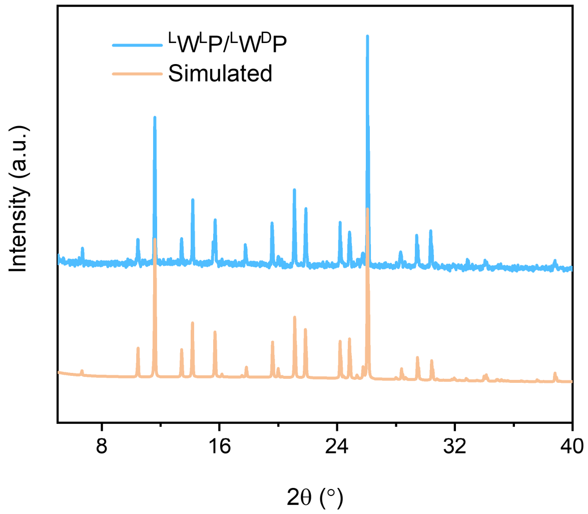

Supplementary Figure 50: Powder X-ray diffraction pattern of c-$^{L}W^{L}P$/c-$^{L}W^{D}P$ co-assemblies.

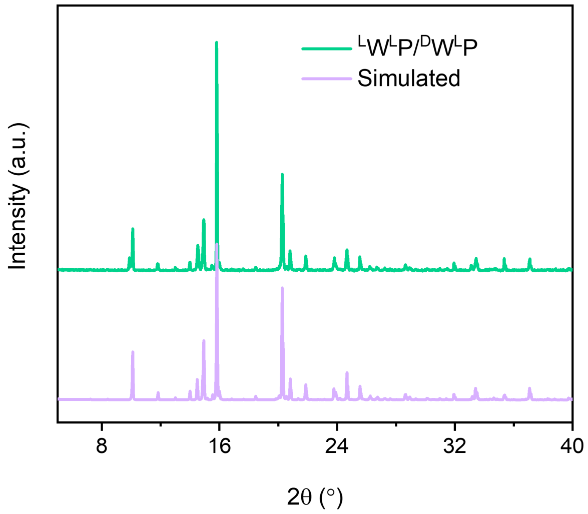

Supplementary Figure 51: Powder X-ray diffraction pattern of c-$^{L}W^{L}P$/c-$^{D}W^{L}P$ co-assemblies.

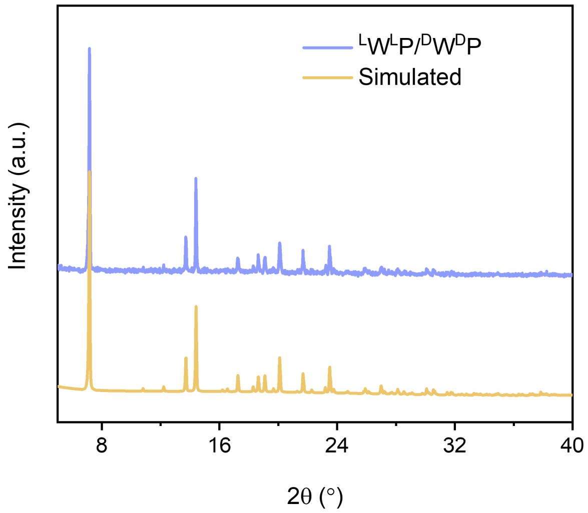

Supplementary Figure 52: Powder X-ray diffraction pattern of c-$^{L}W^{L}P$/c-$^{D}W^{D}P$ co-assemblies.

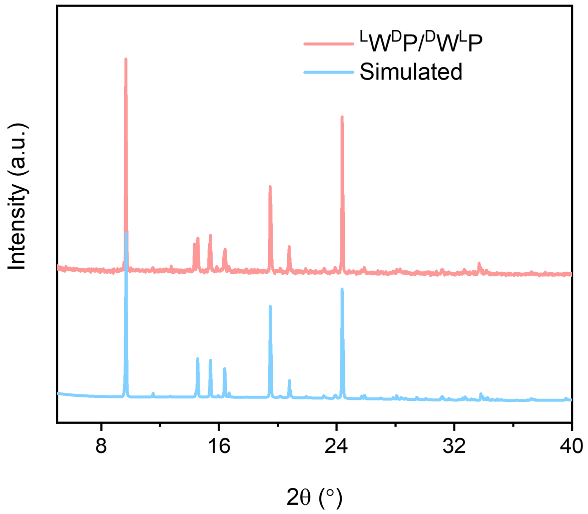

Supplementary Figure 53: Powder X-ray diffraction pattern of c-$^{L}W^{D}P$/c-$^{D}W^{L}P$ co-assemblies.

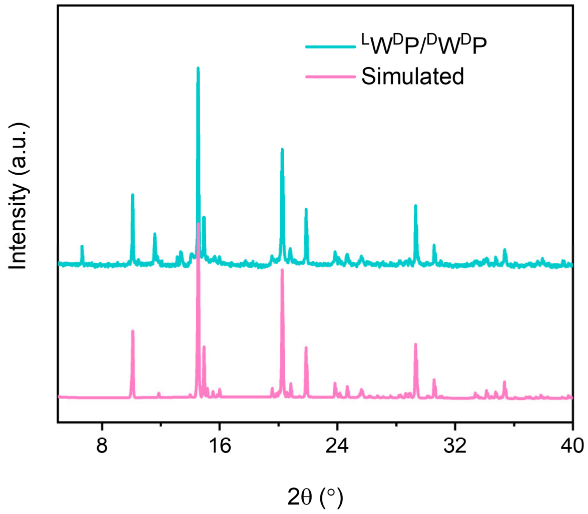

Supplementary Figure 54: Powder X-ray diffraction pattern of c-$^{L}W^{D}P$/c-$^{D}W^{D}P$ co-assemblies.

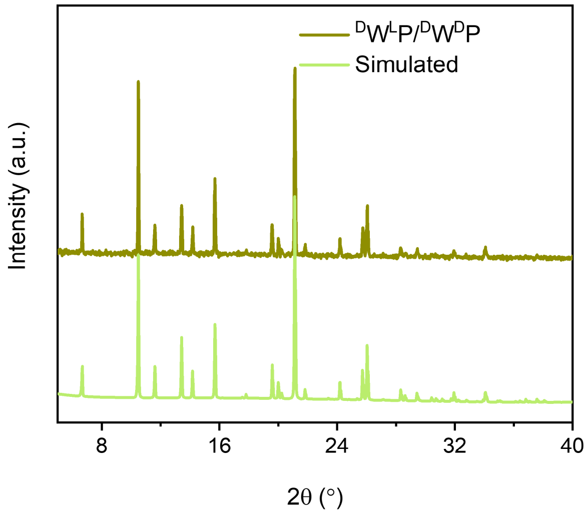

Supplementary Figure 55: Powder X-ray diffraction pattern of c-$^{D}W^{L}P$/c-$^{D}W^{D}P$ co-assemblies.

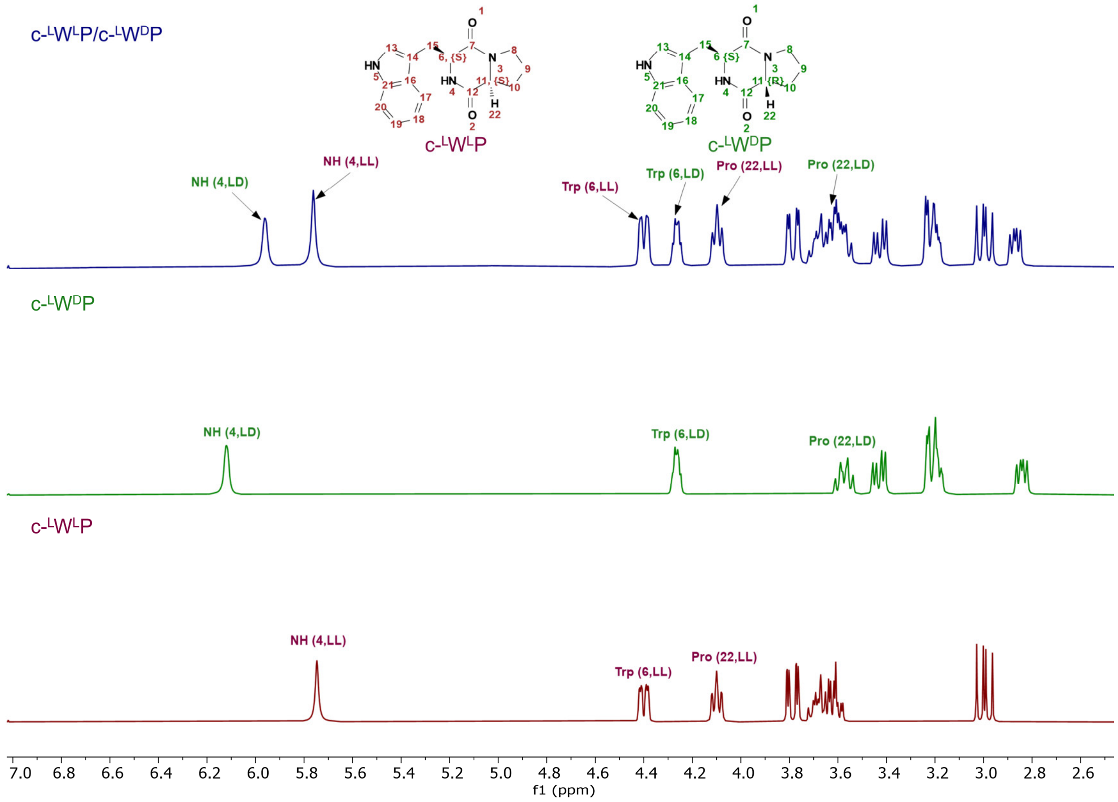

Supplementary Figure 56: Comparative analysis of c-^{L}W^{L}P/c-^{L}W^{D}P co-crystal by experimental ^{1}H NMR (400 MHz) Spectra of c-^{L}W^{L}P (powder), c-^{L}W^{D}P (powder) and c-^{L}W^{L}P/c-^{L}W^{D}P (crystals) in $CDCl_3$ with 1% (v/v) Tetramethylsilane (TMS).

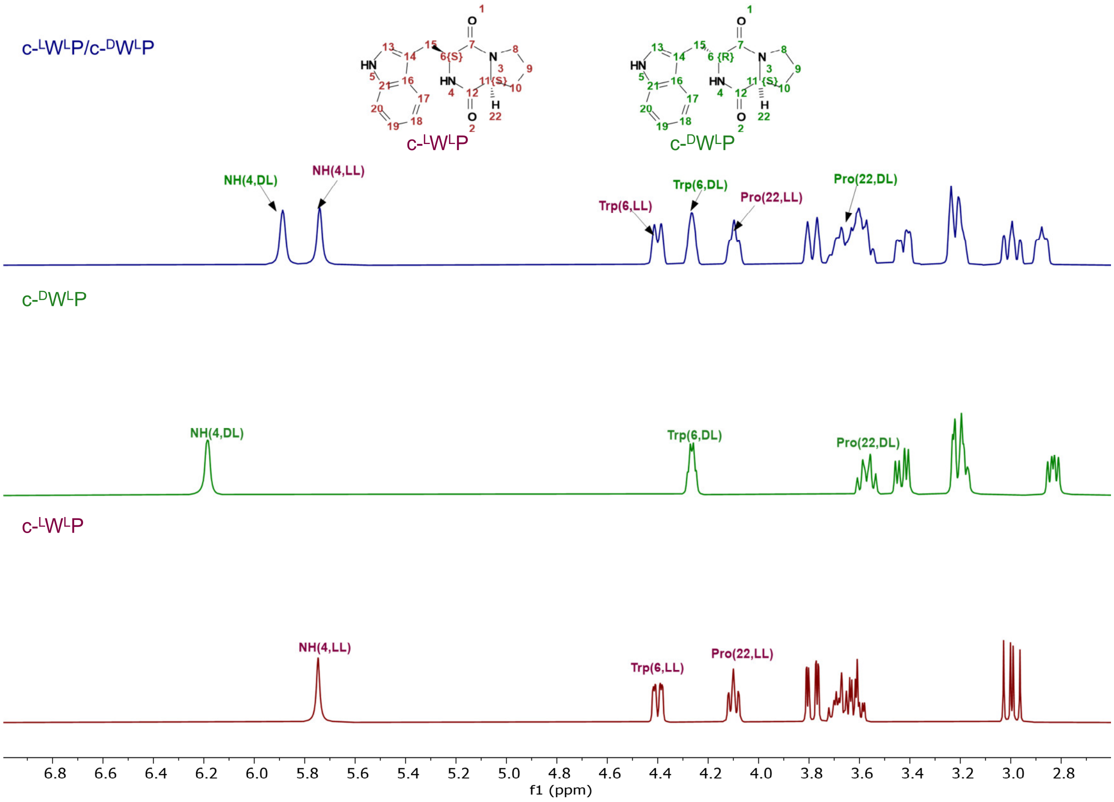

Supplementary Figure 57: Comparative analysis of c-^{L}W^{L}P/^{D}W^{L}P co-crystal by experimental ^{1}H NMR (400 MHz) Spectra of c-^{L}W^{L}P (powder), ^{D}W^{L}P (powder) and c-^{L}W^{L}P/^{D}W^{L}P (crystals) in $CDCl_3$ with 1% (v/v) TMS.

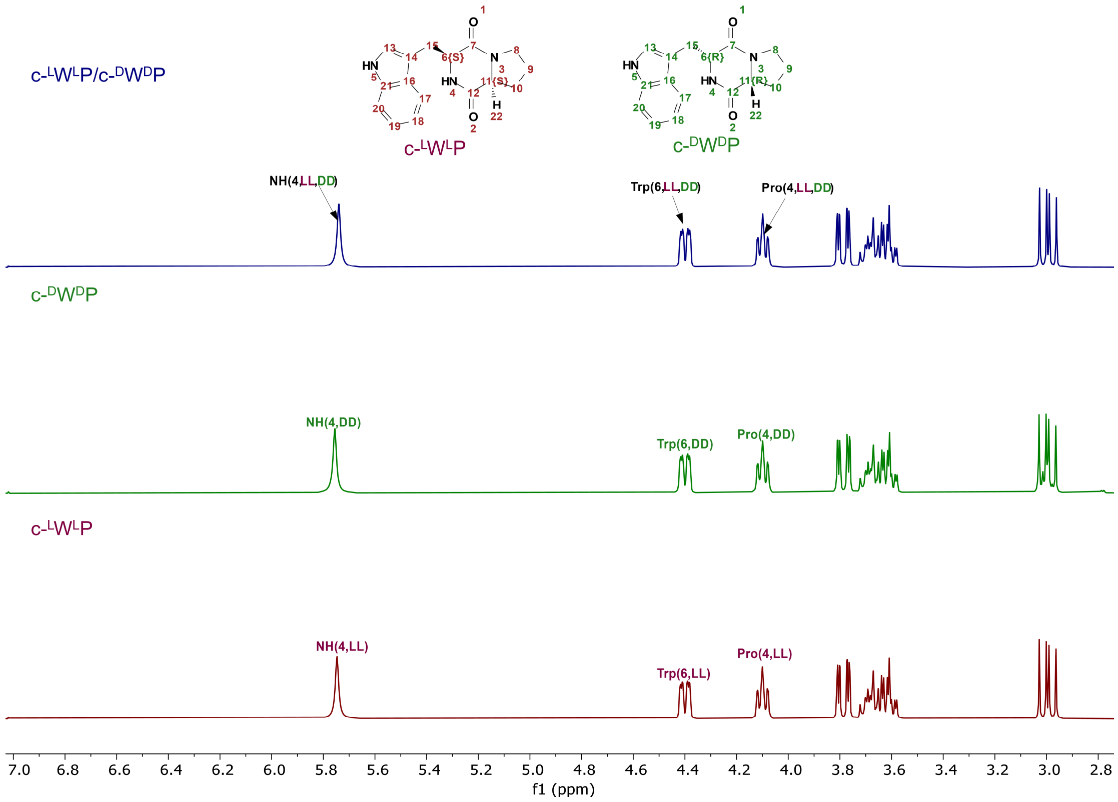

Supplementary Figure 58: Comparative analysis of c-^{L}W^{L}P/c-^{D}W^{D}P co-crystal by experimental ^{1}H NMR (400 MHz) Spectra of c-^{L}W^{L}P (powder), c-^{D}W^{D}P (powder) and c-^{L}W^{L}P/c-^{D}W^{D}P (crystals) in $CDCl_3$ with 1% (v/v) TMS.

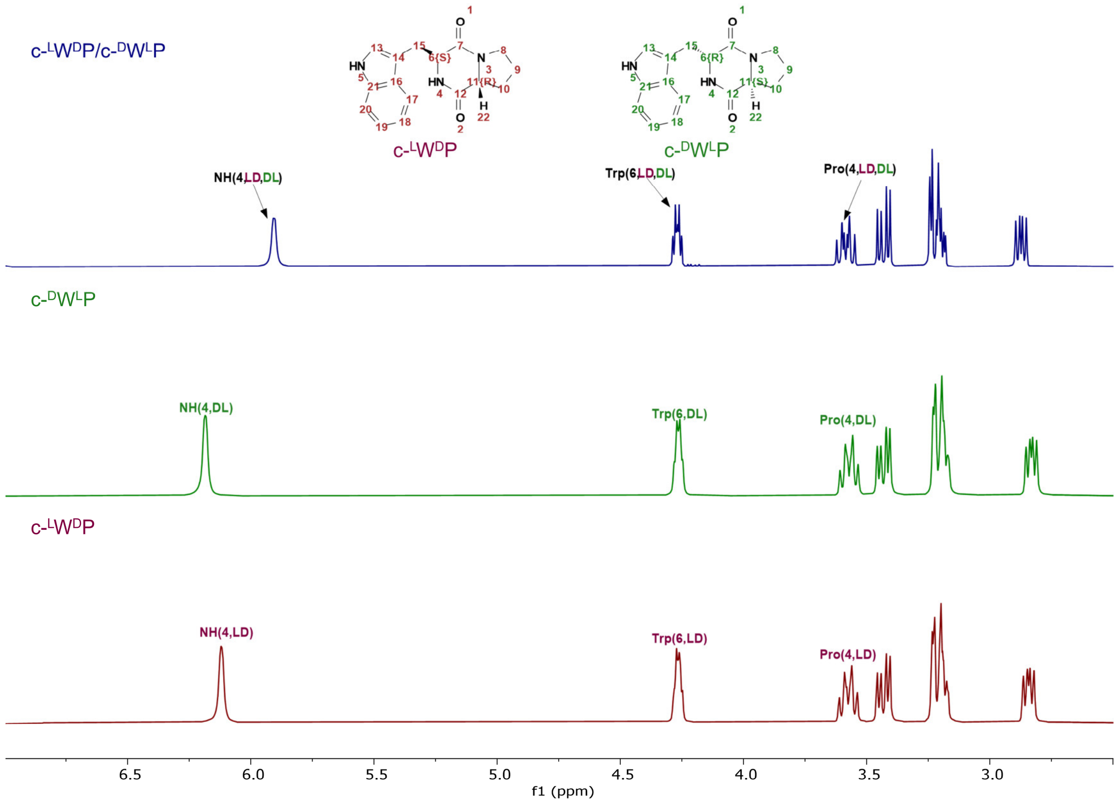

Supplementary Figure 59: Comparative analysis of c-^{L}W^{D}P/c-^{D}W^{L}P co-crystal using experimental ^{1}H NMR (400 MHz) Spectra of c-^{L}W^{D}P (powder), c-^{D}W^{L}P (powder) and c-^{L}W^{D}P/c-^{D}W^{L}P (crystals) in $CDCl_3$ with 1% (v/v) TMS.

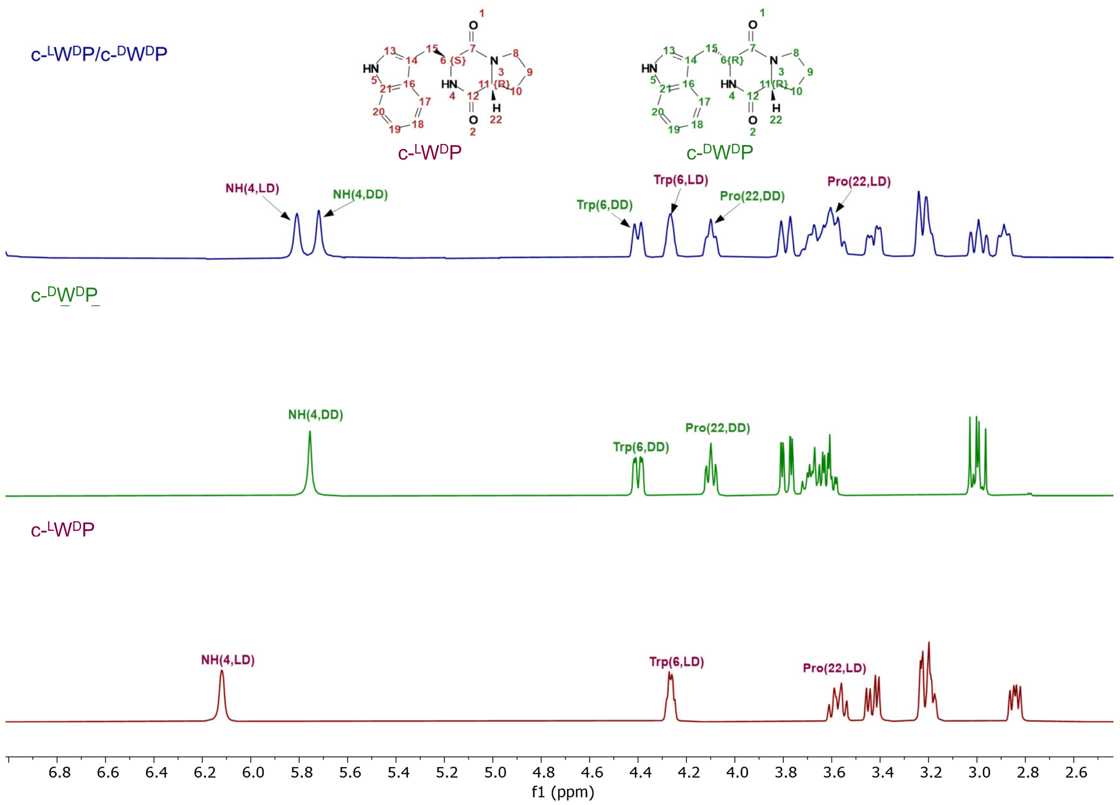

Supplementary Figure 60: Comparative analysis of c-^{L}W^{D}P/c-^{D}W^{D}P co-crystal using experimental ^{1}H NMR (400 MHz) Spectra of c-^{L}W^{D}P (powder), c-^{D}W^{D}P (powder) and c-^{L}W^{D}P/c-^{D}W^{D}P (crystals) in $CDCl_3$ with 1% (v/v) TMS.

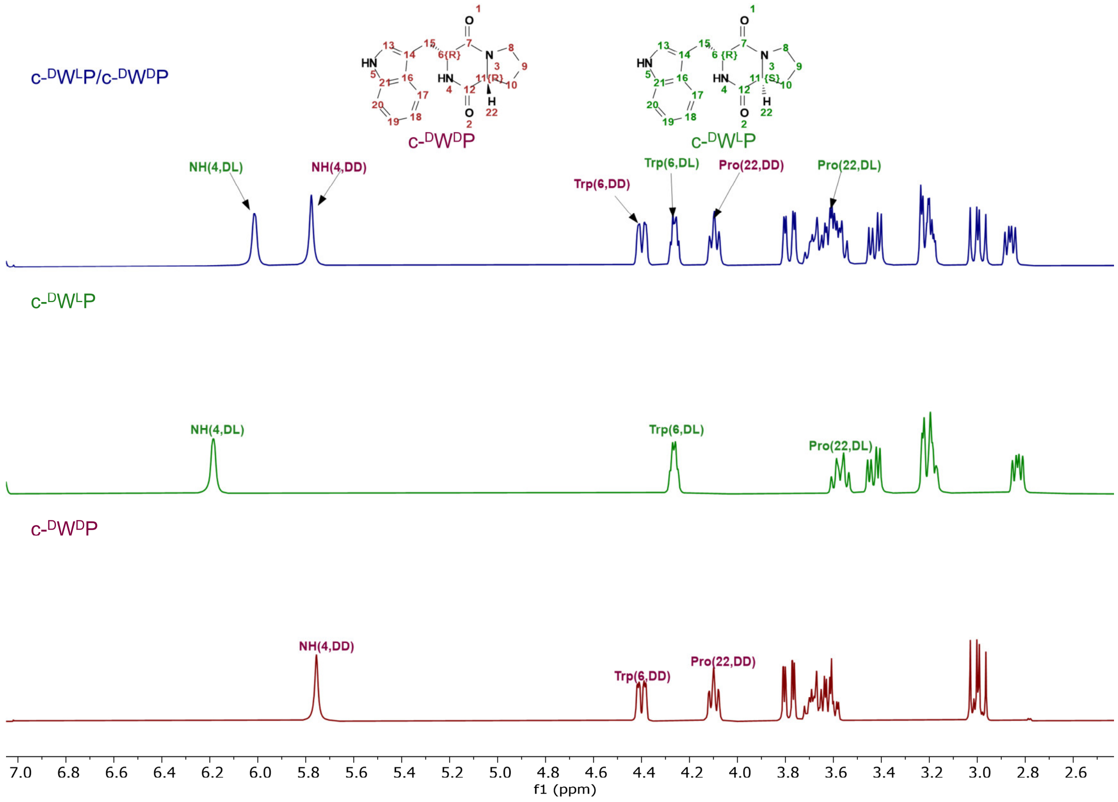

Supplementary Figure 61: Comparative analysis of c-^{D}W^{L}P/c-^{D}W^{D}P co-crystal using experimental ^{1}H NMR (400 MHz) Spectra of c-^{D}W^{L}P (powder), c-^{D}W^{D}P (powder) and c-^{D}W^{L}P/c-^{D}W^{D}P (crystals) in $CDCl_3$ with 1% (v/v) TMS.

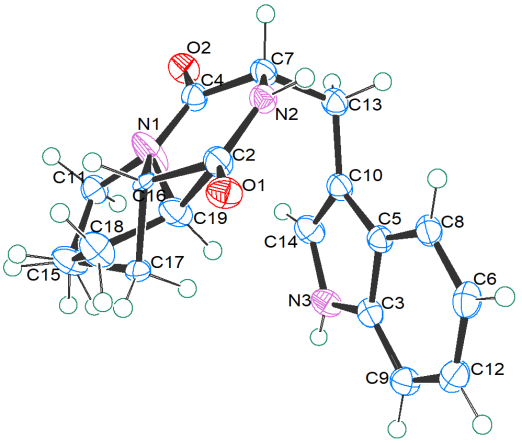

Supplementary Figure 62: ORTEP diagram of the c-^{L}W^{L}P/c-^{L}W^{D}P co-crystal with ellipsoid probability of 50%. CCDC ref. no. 2465116.

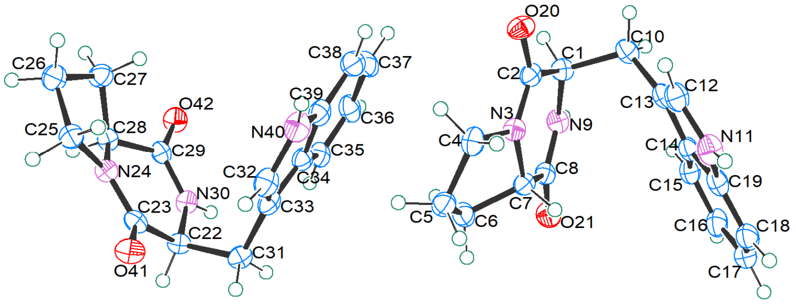

Supplementary Figure 63: ORTEP diagram of the c-^{L}W^{L}P/c-^{D}W^{L}P co-crystal with ellipsoid probability of 50%. CCDC ref. no. 2465117.

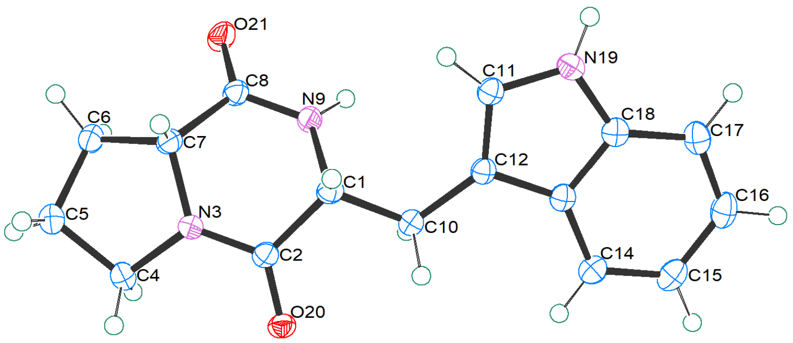

Supplementary Figure 64: ORTEP diagram of the c-^{L}W^{L}P/c-^{D}W^{D}P co-crystal with ellipsoid probability of 50%. CCDC ref. no. 2465118.

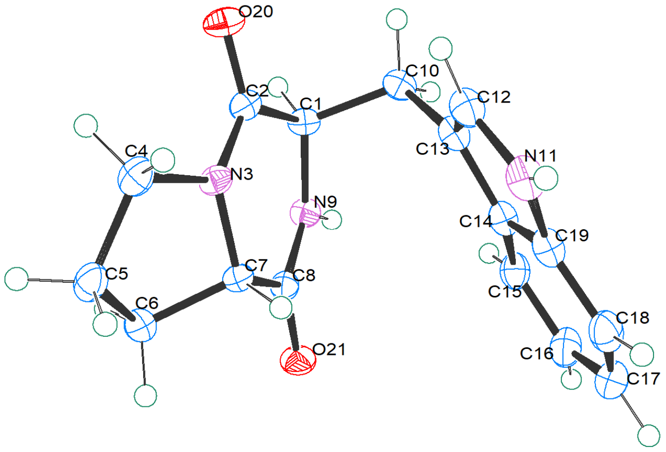

Supplementary Figure 65: ORTEP diagram of the c-^{L}W^{D}P/c-^{D}W^{L}P co-crystal with ellipsoid probability of 50%. CCDC ref. no. 2465119.

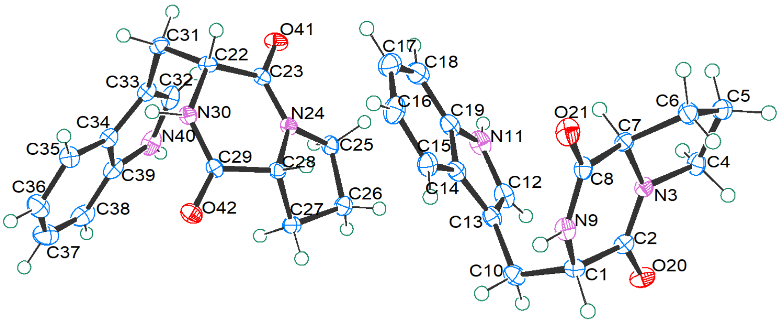

Supplementary Figure 66: ORTEP diagram of the c-^{L}W^{D}P/c-^{D}W^{D}P co-crystal with ellipsoid probability of 50%. CCDC ref. no. 2465120.

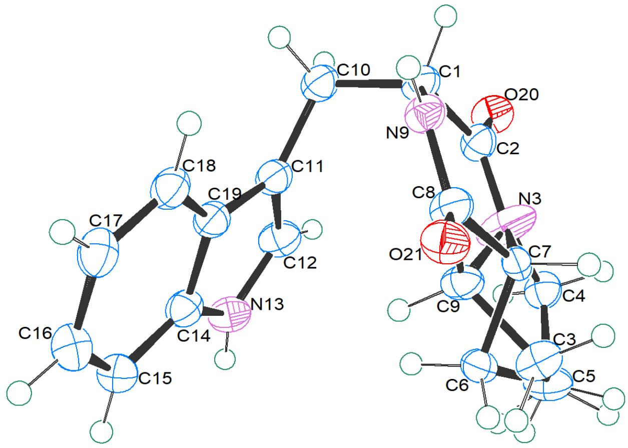

Supplementary Figure 67: ORTEP diagram of the c-^{D}W^{L}P/c-^{D}W^{D}P co-crystal with ellipsoid probability of 50%. CCDC ref. no. 2465121.

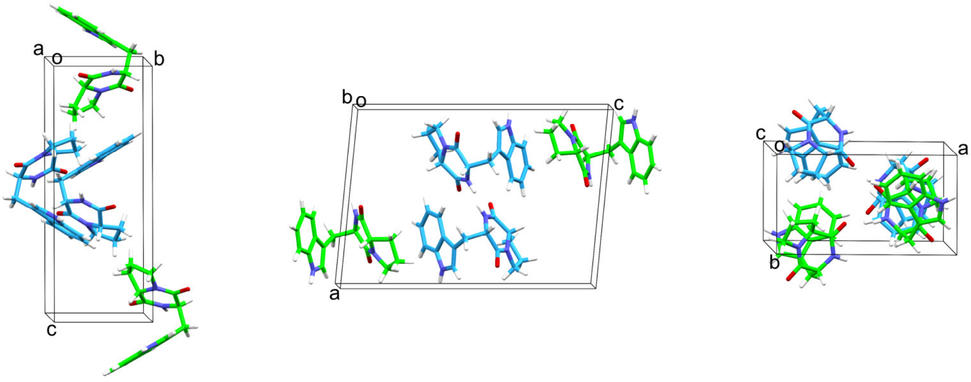

Supplementary Figure 68: Unit cell of c-^{L}W^{L}P/c-^{D}W^{L}P co-assemblies visualized along different directions.

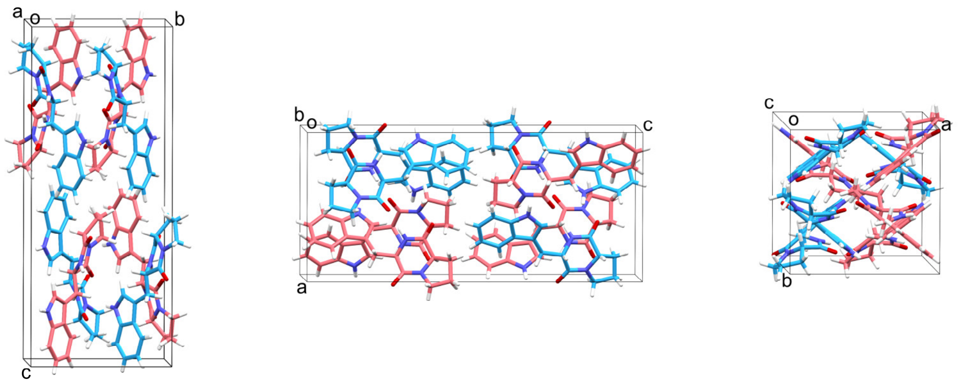

Supplementary Figure 69: Unit cell of c-^{L}W^{L}P/c-^{D}W^{D}P co-assemblies visualized along different directions.

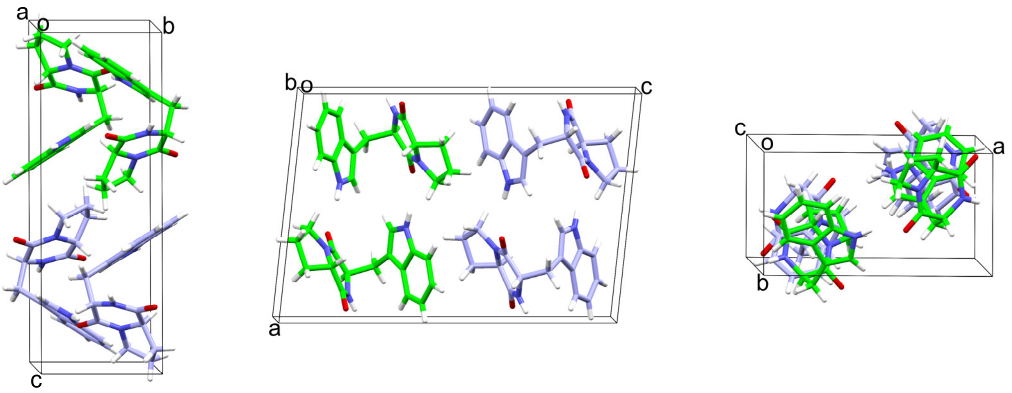

Supplementary Figure 70: Unit cell of c-^{L}W^{D}P/c-^{D}W^{L}P co-assemblies visualized along different directions.

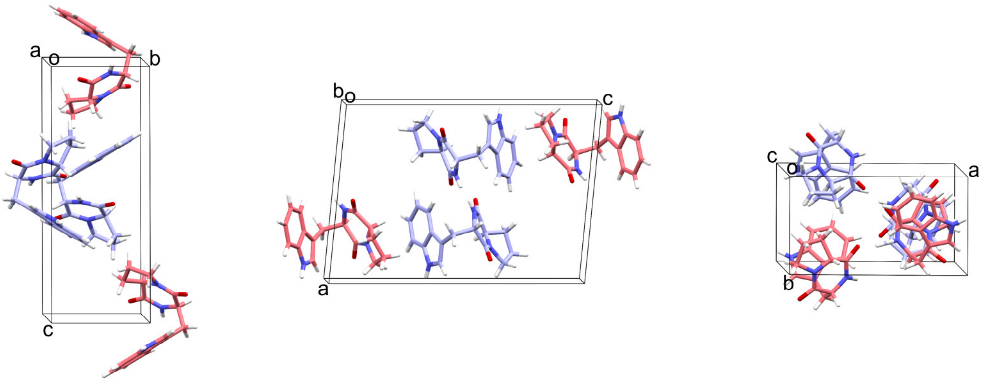

Supplementary Figure 71: Unit cell of c-^{L}W^{D}P/c-^{D}W^{D}P co-assemblies visualized along different directions.

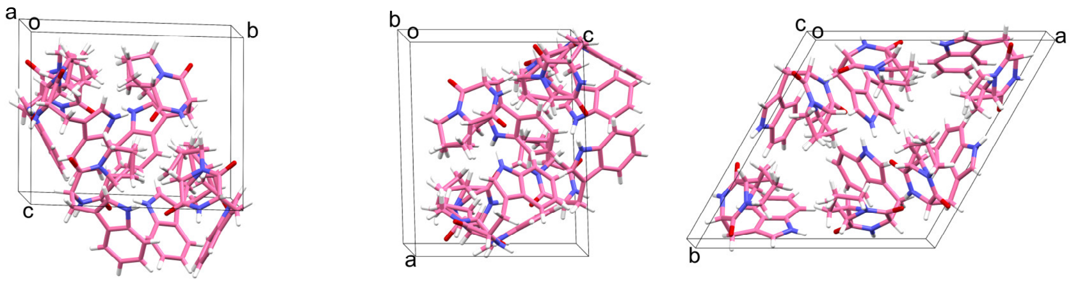

Supplementary Figure 72: Unit cell of c-^{L}W^{L}P/c-^{L}W^{D}P co-assemblies visualized along different directions.

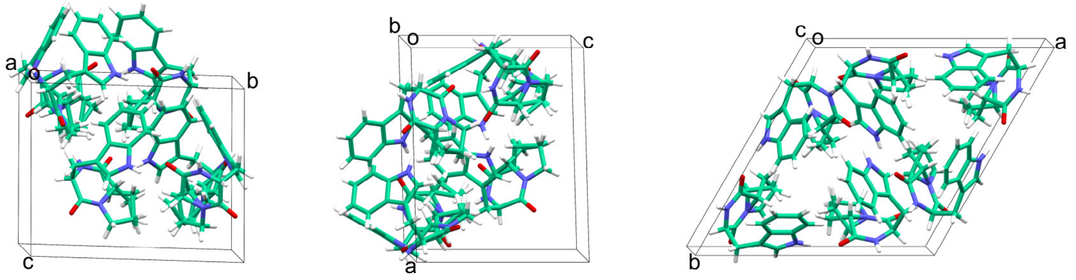

Supplementary Figure 73: Unit cell of c-^{D}W^{L}P/c-^{D}W^{D}P co-assemblies visualized along different directions.

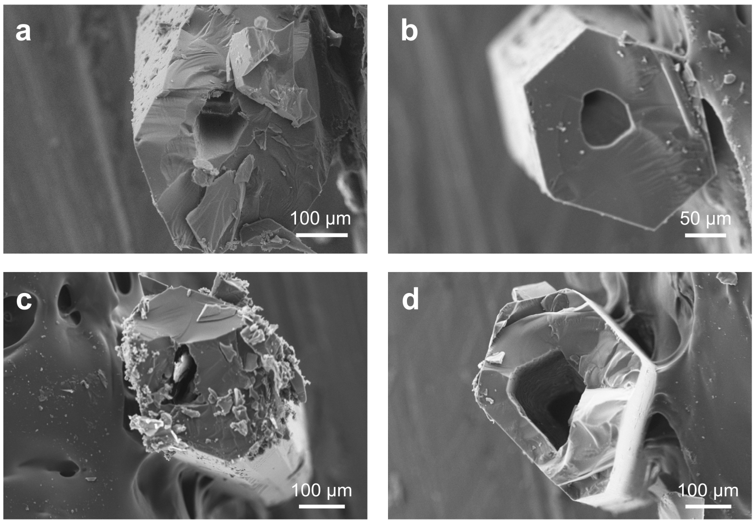

Supplementary Figure 74: SEM images of (a) c-$^{L}W^{L}P$, (b) c-$^{L}W^{D}P$, (c) c-$^{D}W^{L}P$, and (d) c-$^{D}W^{D}P$ assemblies showing pore structures.

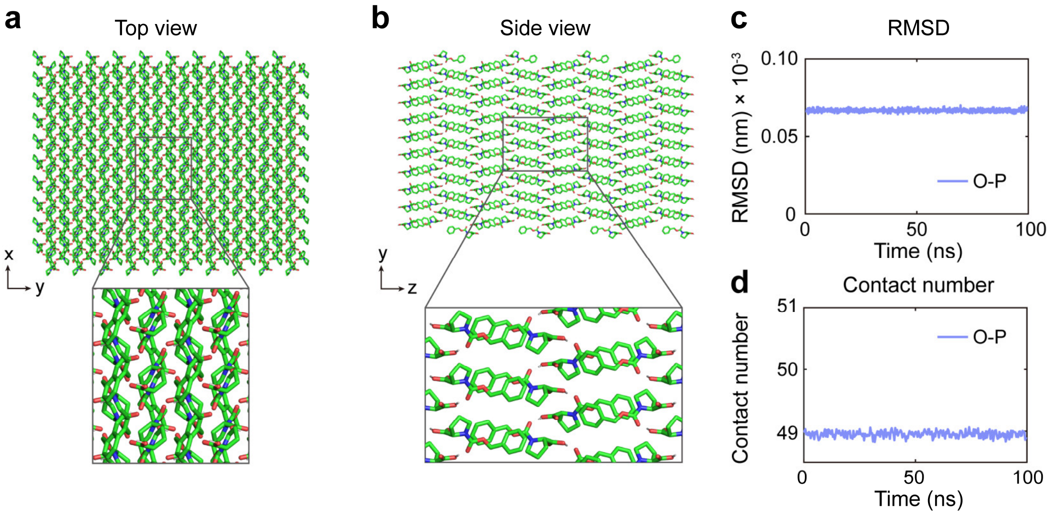

Supplementary Figure 75: The molecular packing and structural stability of the O-P crystal. (a-b) The initial conformation of MD simulations in two different views of a 10 × 10 × 6 supercell crystal structure: (a) top view and (b) side view. The detailed molecular packings in the crystal structure are highlighted by enlarged views. (c-d) Time evolution of (c) all-atom RMSD of O-P molecules and (d) inter-molecular contact number of the crystal. The RMSD and the inter-molecular contact numbers of each system remain stable over the 100 ns simulation time, confirming the stability of the constructed crystal structures.

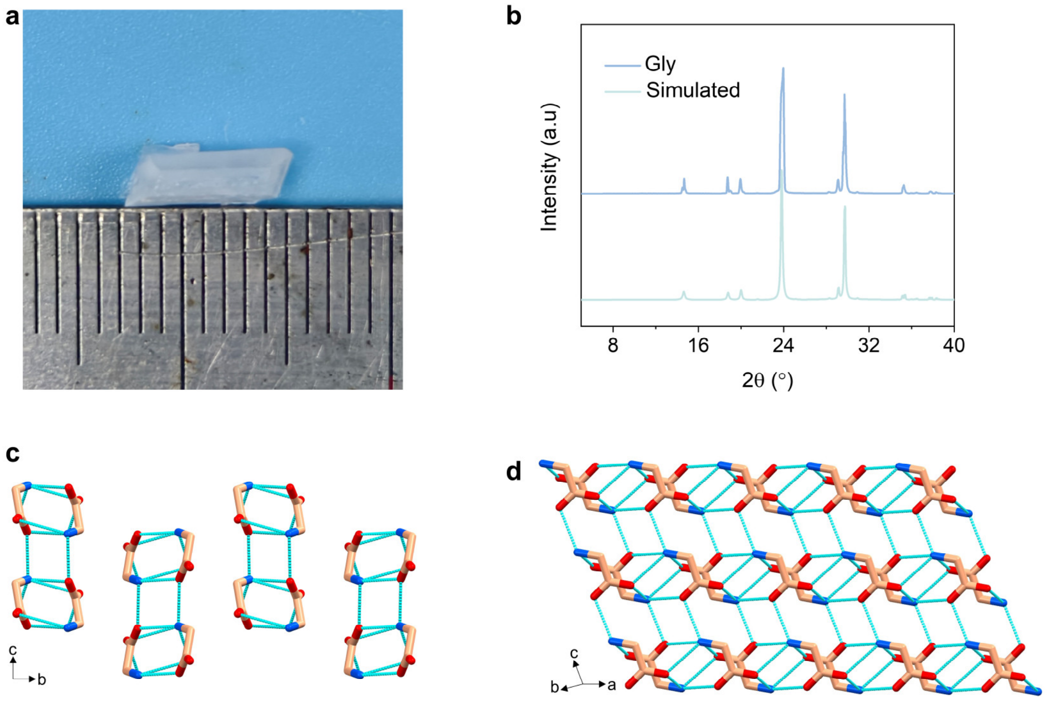

Supplementary Figure 76: (a) Photograph of a Gly self-assembled crystal. (b) Powder X-ray diffraction pattern showing that the Gly assemblies belong to the α-Gly structure. (c,d) Gly molecules packed into a layered supramolecular structure. CCDC ref. no. 193596[1].

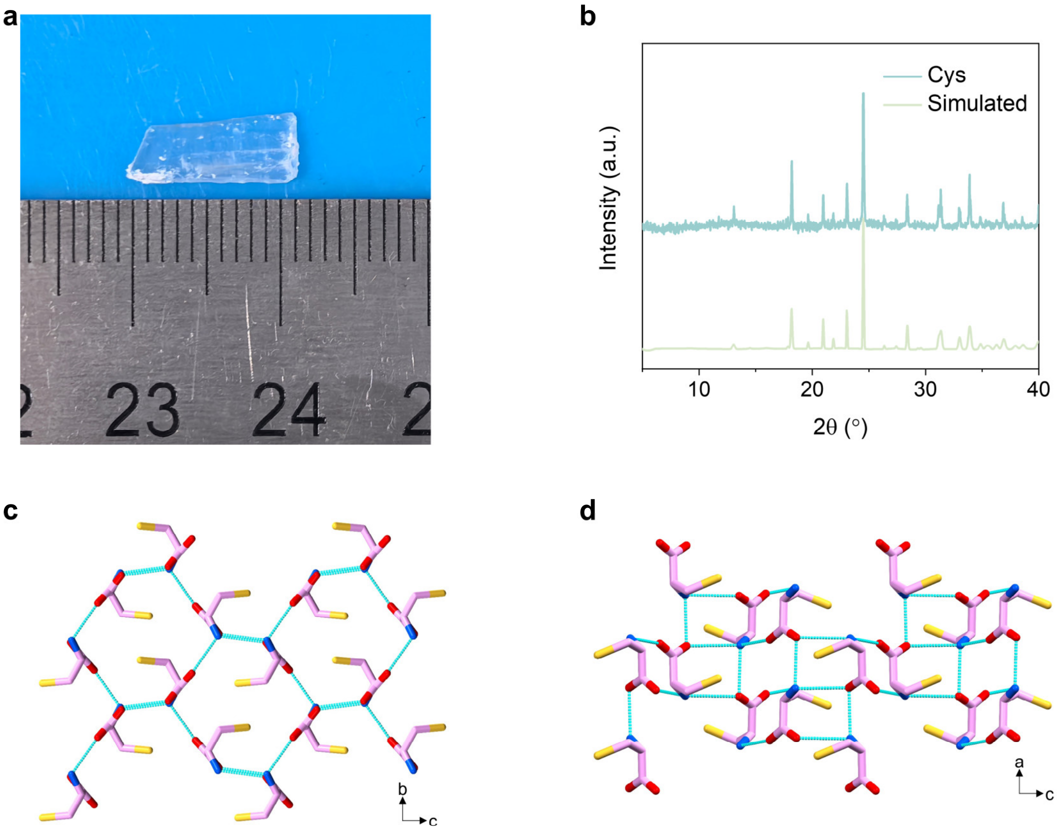

Supplementary Figure 77: (a) Photograph of a Cys self-assembled crystal. (b) Powder X-ray diffraction pattern of Cys assemblies. (c,d) Cys molecules interconnected by 3D hydrogen-bond networks. CCDC ref. no. 683376[2].

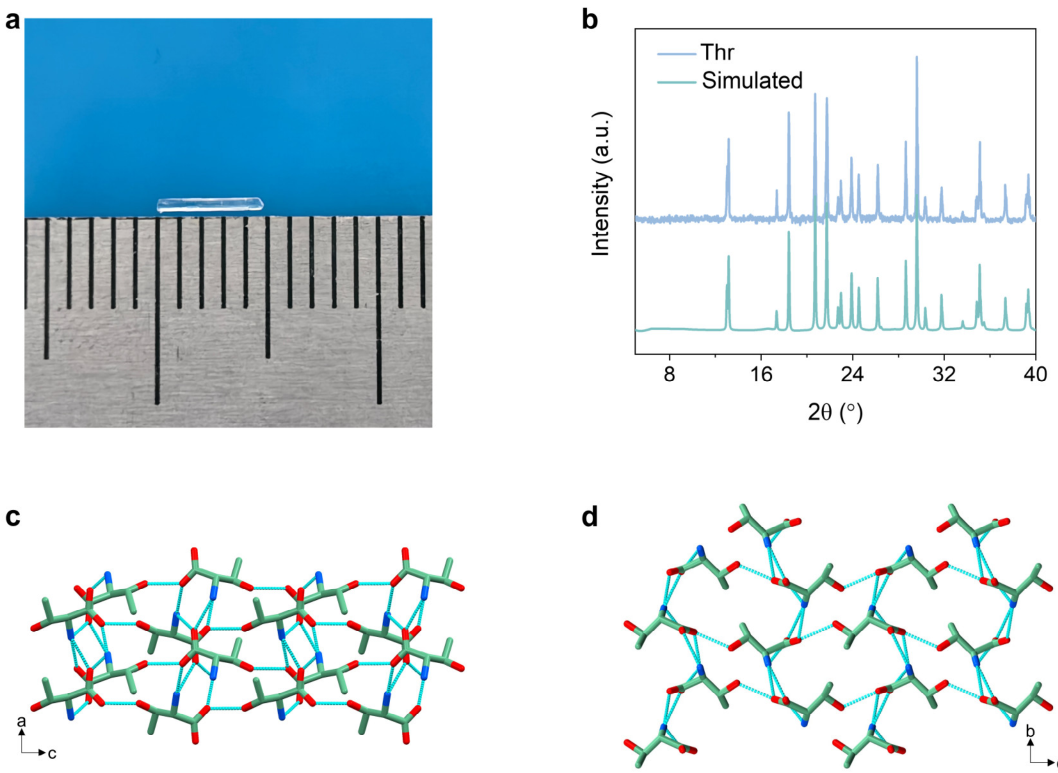

Supplementary Figure 78: (a) Photograph of a Thr self-assembled crystal. (b) Powder X-ray diffraction pattern of Thr assemblies. (c,d) Thr molecules interconnected by dense 3D hydrogen-bond networks. CCDC ref. no. 2024959[3].

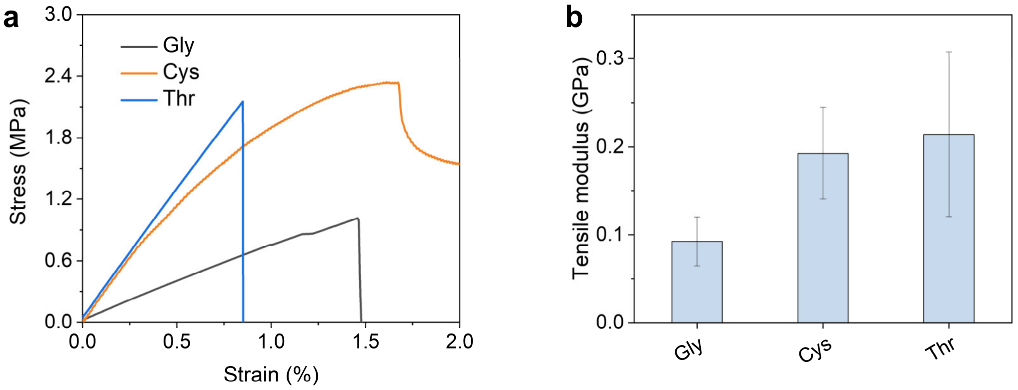

Supplementary Figure 79: (a) Typical stress-strain curves of Gly, Cys, and Thr crystals under tension. (b) Comparison of tensile modulus obtained from Gly, Cys, and Thr crystals. Error bars for tensile modulus represent the standard deviation from five independent measurements.

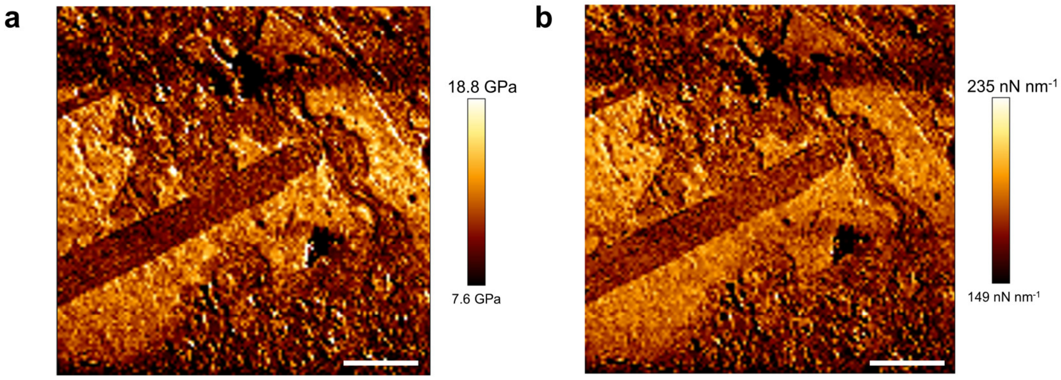

Supplementary Figure 80: (a) Topographic Young's modulus maps, and (b) Topographic point stiffness maps of c-^{L}W^{L}P, Scale bar = 1 μm.

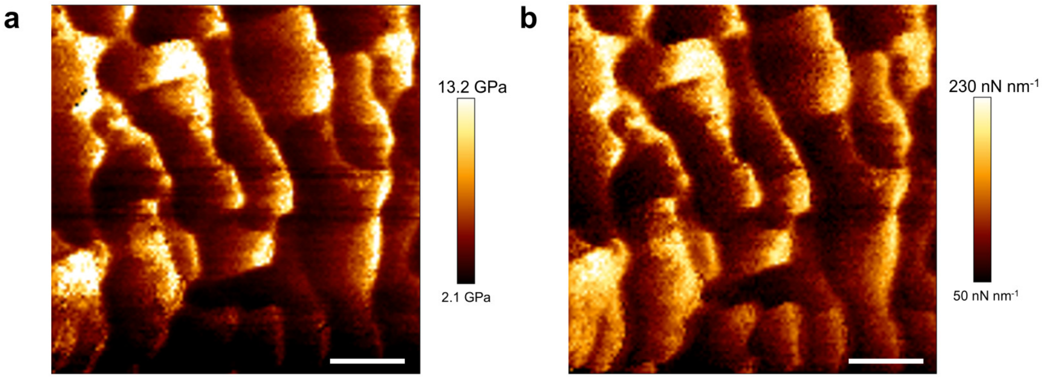

Supplementary Figure 81: (a) Topographic Young's modulus map, and (b) topographic point stiffness map of c-^{L}W^{D}P, Scale bar = 1 μm.

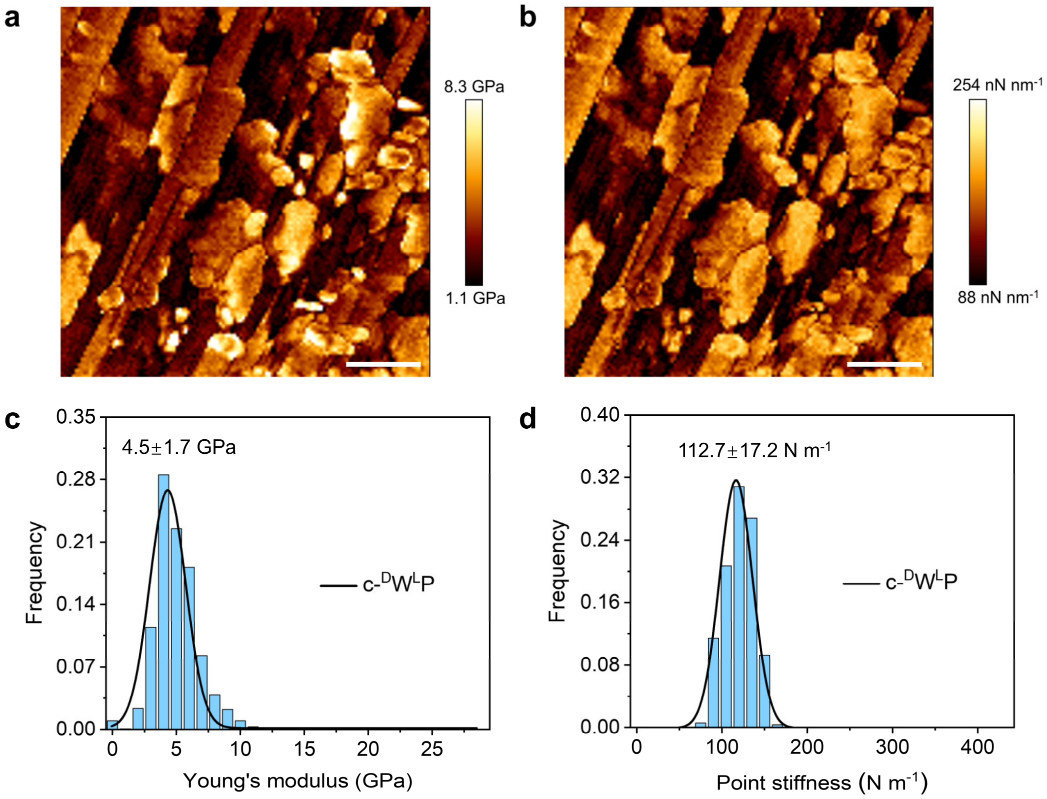

Supplementary Figure 82: (a) Topographic Young's modulus map, (b) topographic point stiffness map, (c) statistical Young's modulus distribution, and (d) statistical point stiffness distribution of c-^{D}W^{L}P, Scale bar = 1 µm.

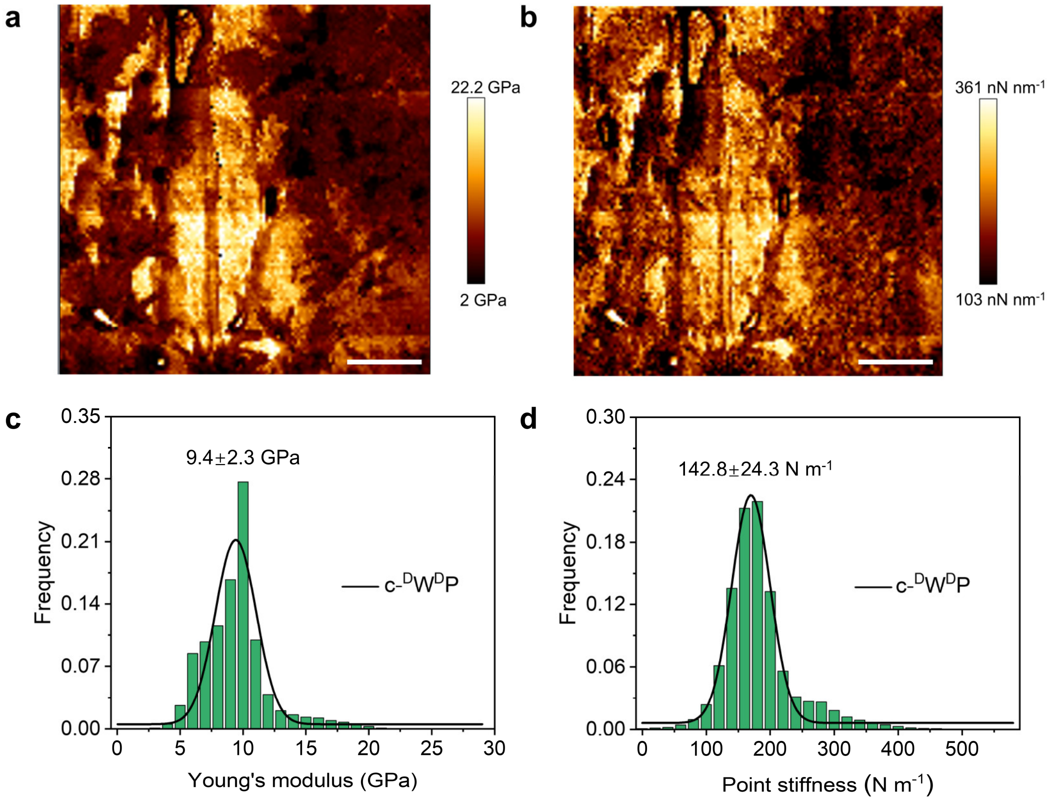

Supplementary Figure 83: (a) Topographic Young's modulus map, (b) topographic point stiffness map, (c) statistical Young's modulus distribution, and (d) statistical point stiffness distribution of c-$^{D}W^{D}P$, Scale bar = 1 μm.

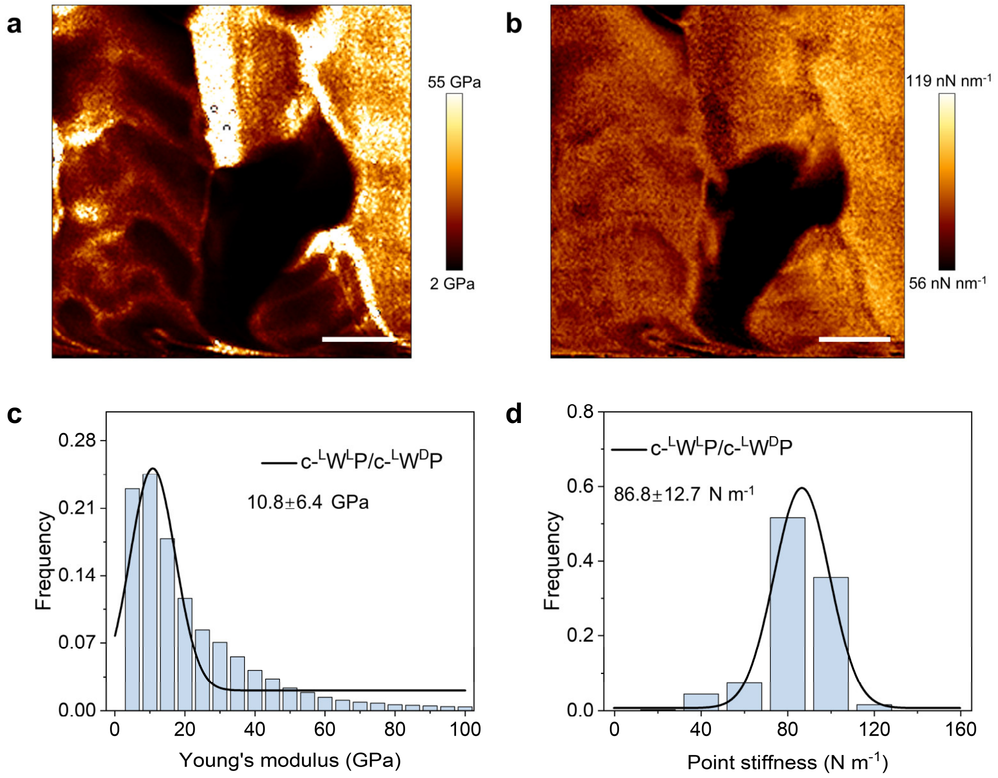

Supplementary Figure 84: (a) Topographic Young's modulus map, (b) topographic point stiffness map, (c) statistical Young's modulus distribution, and (d) statistical point stiffness distribution of c-^{L}W^{L}P/c-^{L}W^{D}P, Scale bar = 1 μm.

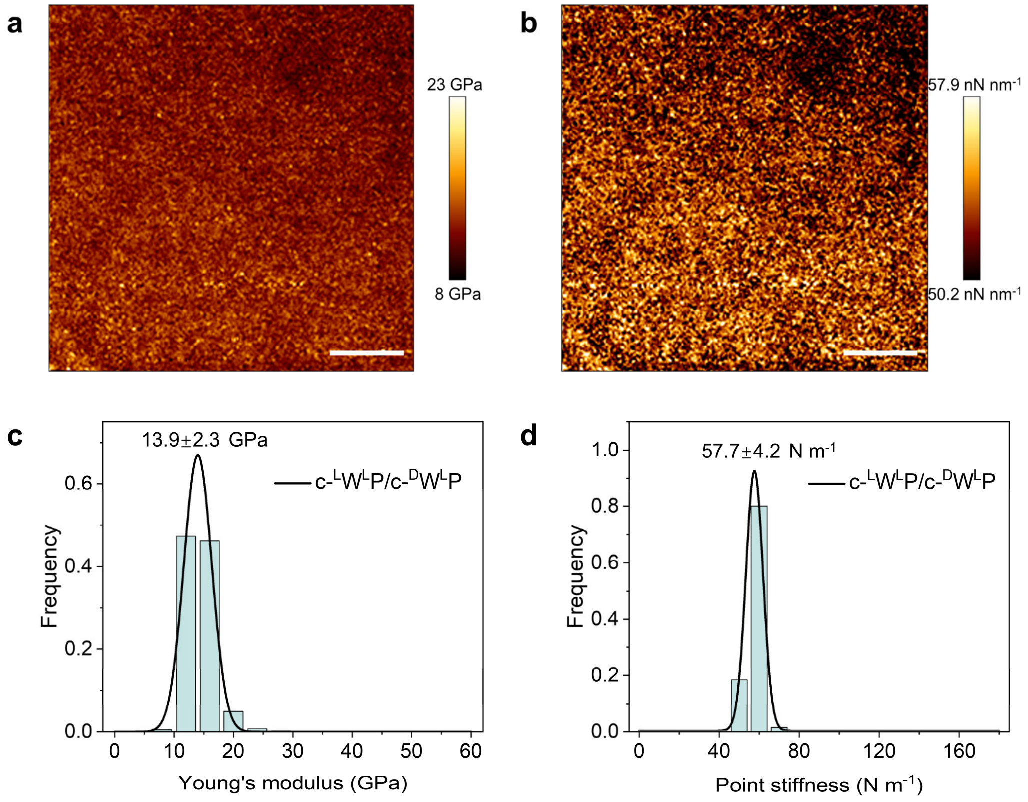

Supplementary Figure 85: (a) Topographic Young's modulus map, (b) topographic point stiffness map, (c) statistical Young's modulus distribution, and (d) statistical point stiffness distribution of c-$^{L}W^{L}P$/c-$^{D}W^{L}P$, Scale bar = 1 μm.

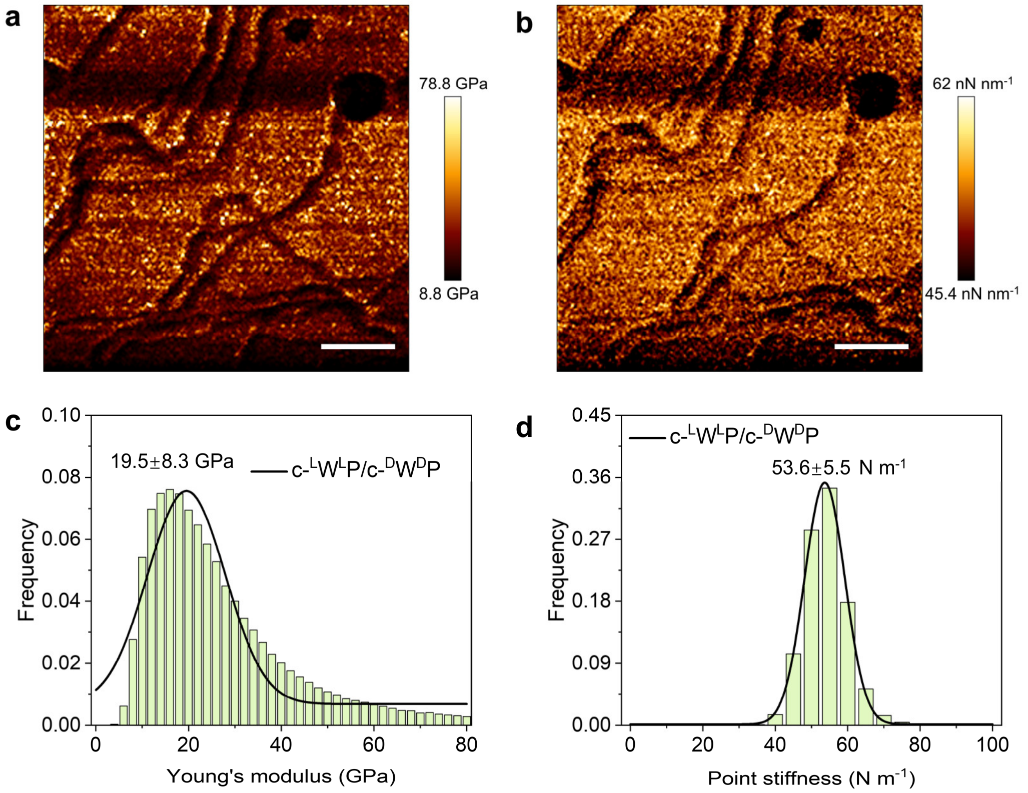

Supplementary Figure 86: (a) Topographic Young's modulus map, (b) topographic point stiffness map, (c) statistical Young's modulus distribution, and (d) statistical point stiffness distribution of c-^{L}W^{L}P/c-^{D}W^{D}P, Scale bar = 1 μm.

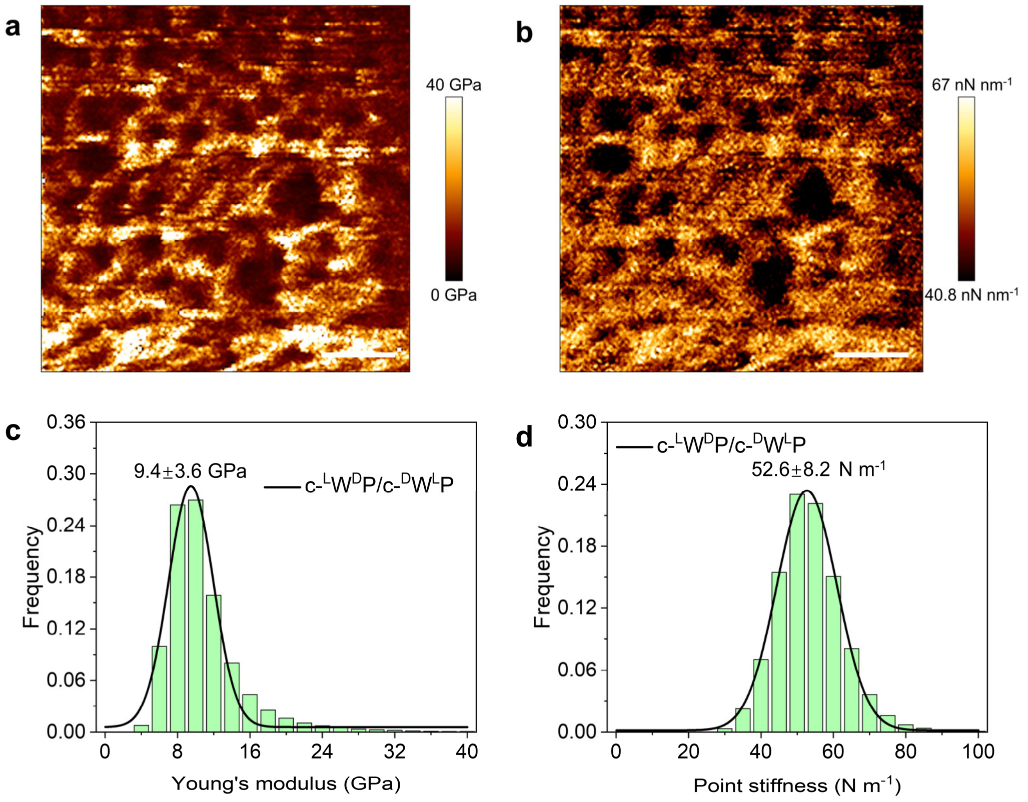

Supplementary Figure 87: (a) Topographic Young's modulus map, (b) topographic point stiffness map, (c) statistical Young's modulus distribution, and (d) statistical point stiffness distribution of c-$^{L}W^{D}P$/c-$^{D}W^{L}P$, Scale bar = 1 μm.

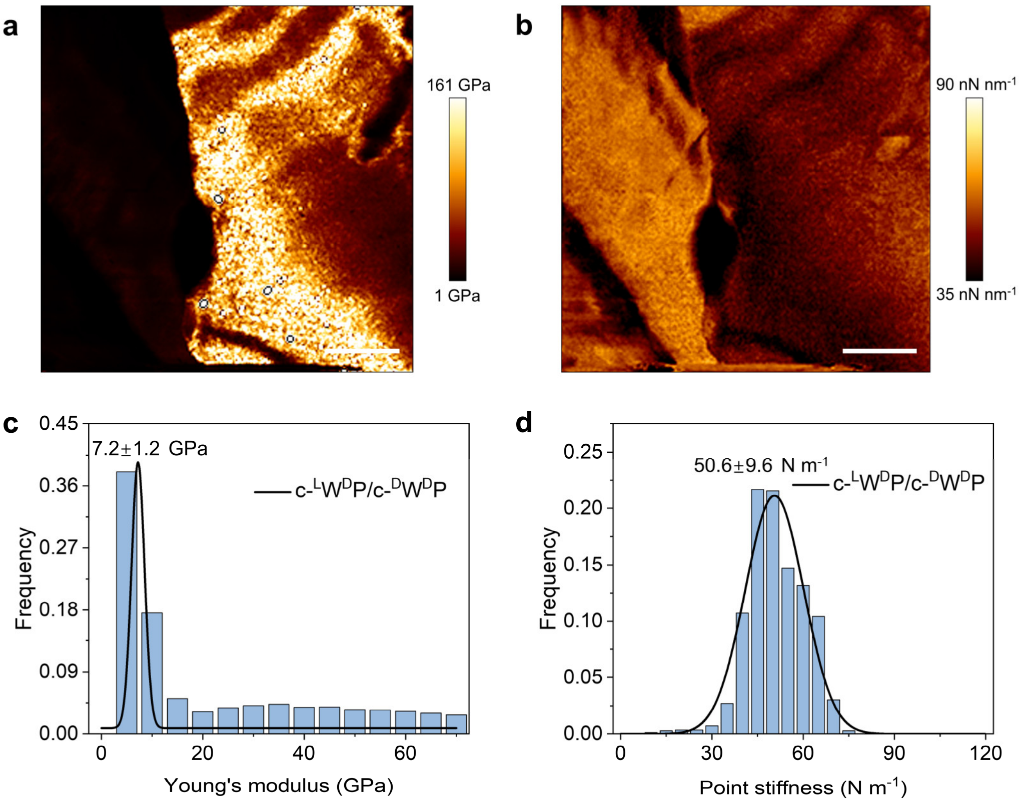

Supplementary Figure 88: (a) Topographic Young's modulus map, (b) topographic point stiffness map, (c) statistical Young's modulus distribution, and (d) statistical point stiffness distribution of c-^{L}W^{D}P/c-^{D}W^{D}P, Scale bar = 1 μm.

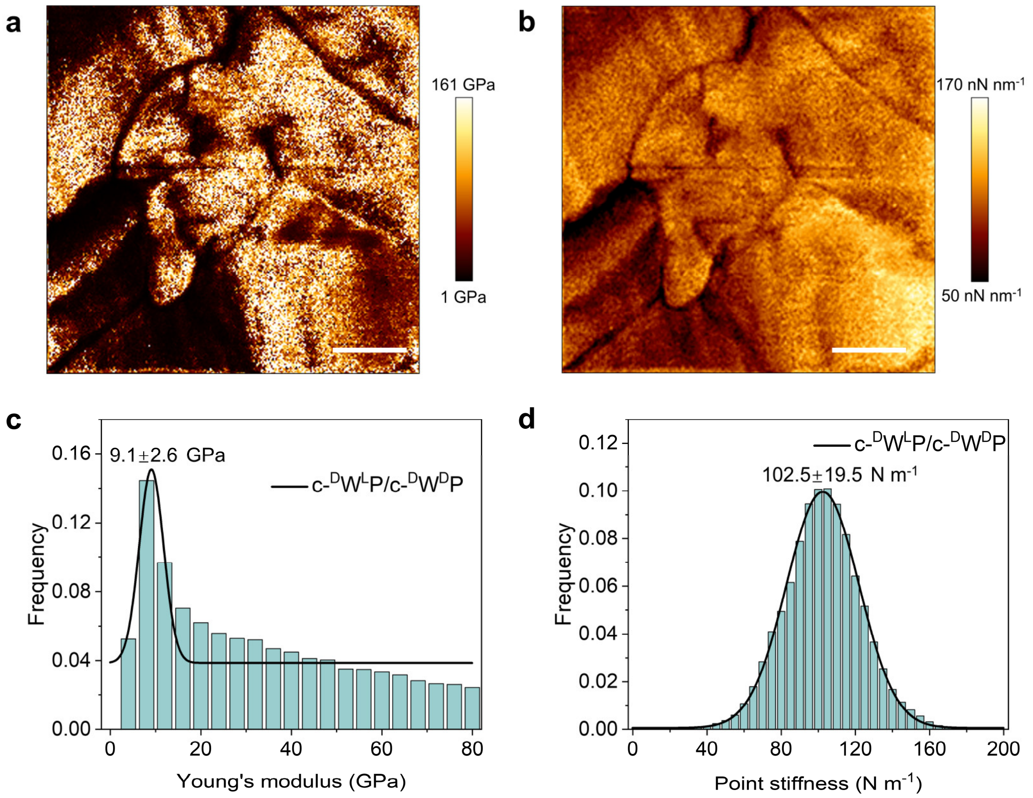

Supplementary Figure 89: (a) Topographic Young's modulus map, (b) topographic point stiffness map, (c) statistical Young's modulus distribution, and (d) statistical point stiffness distribution of c-^{D}W^{L}P/c-^{D}W^{D}P, Scale bar = 1 μm.

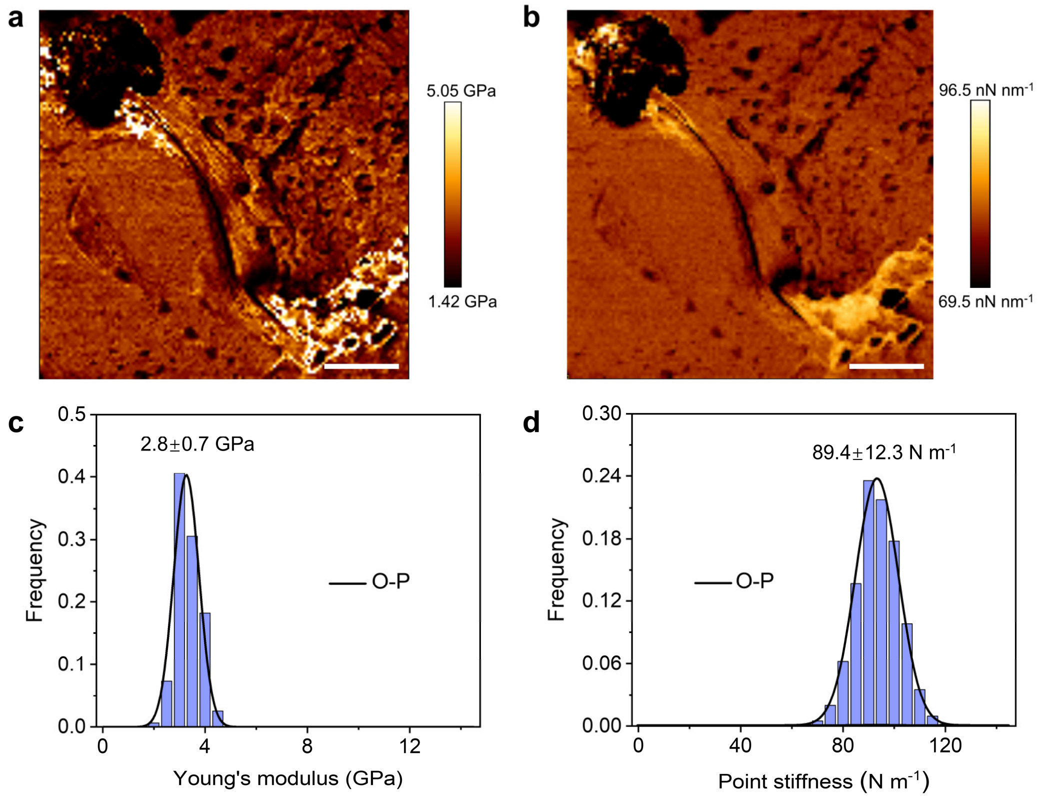

Supplementary Figure 90: (a) Topographic Young's modulus map, (b) topographic point stiffness map, (c) statistical Young's modulus distribution, and (d) statistical point stiffness distribution of O-P, Scale bar = 1 μm.

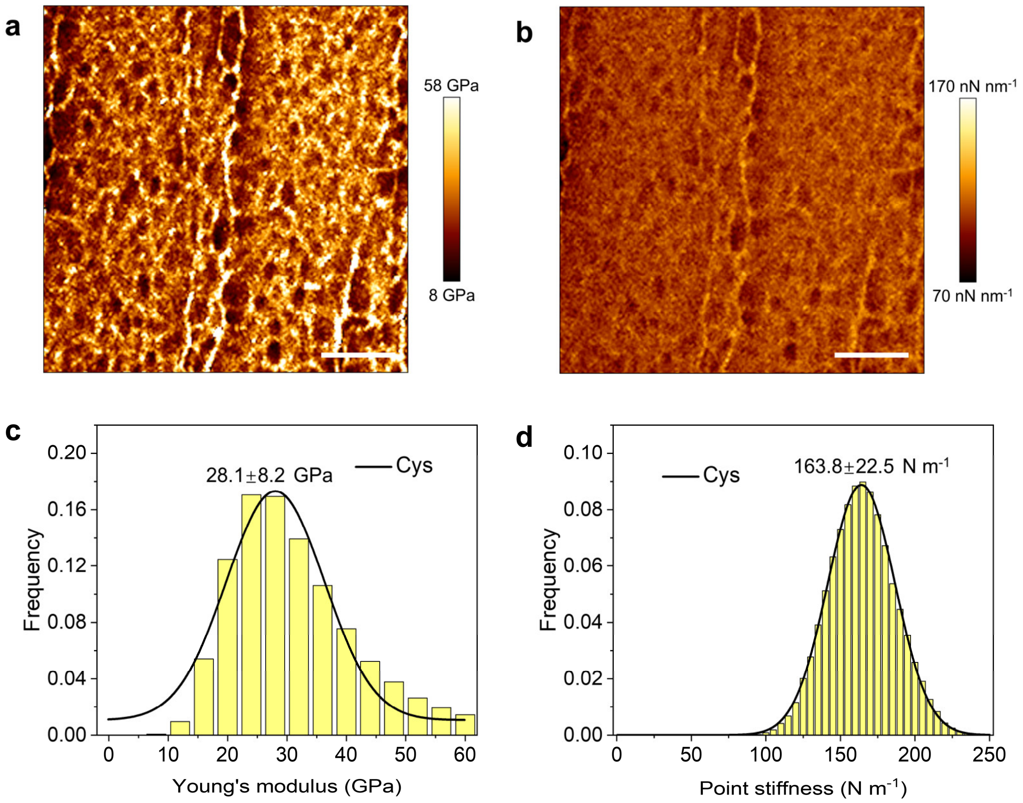

Supplementary Figure 91: (a) Topographic Young's modulus map, (b) topographic point stiffness map, (c) statistical Young's modulus distribution, and (d) statistical point stiffness distribution of Cys. Scale bar = 1 μm.

Supplementary Table 1: Data collection and refinement statistics of c-$^{L}W^{L}P$ and c-$^{L}W^{D}P$ crystals.

**Crystal data**	**c-$^{L}W^{L}P$**	**c-$^{L}W^{D}P$**
**CCDC Deposition number**	2465098	2465099
**Chemical formula**	$C_{16}H_{17}N_3O_2$	$C_{16}H_{17}N_3O_{2.67}$
**Crystal system**	Hexagonal	Hexagonal
**Space group**	$P\ 6_3$	$P\ 6_3$
**Mr**	283.32	293.99
**Volume (Å^3)**	2093.83(3)	2158.90(3)
**a(Å)**	14.9474(1)	15.1447(10)
**b (Å)**	14.9474(1)	15.1447(10)
**c (Å)**	10.8213(1)	10.8688(10)
**α (°)**	90	90
**β (°)**	90	90
**γ (°)**	120	120
**Z**	6	6
**Mu (mm^{-1})**	0.738	0.772
**Temperature**	120 K	113.15 K
**Data collection Diffractometer**	Rigaku Synergy R HyPix-Arc150 (Cu)	Rigaku XtaLAB P200 Pilatus 007 (Mo)
**No. of reflections(unique)**	332135(3064)	30407(2976)
**θ° range**	3.414 to 79.823°	5.286 to 77.286°
**Reflections/restraints/parameters**	3064/1/190	2976/7/217
**R[F2 > 2σ(F2)]**	0.0304	0.0287
**wR(F2)**	0.0785	0.0794
**Goodness-of-fit**	1.038	1.069
**H-atom treatment**	calc, riding	mixed

Supplementary Table 2: Data collection and refinement statistics of c-$^{D}W^{L}P$ and c-$^{D}W^{D}P$ crystals.

Crystal data	c-$^{D}W^{L}P$	c-$^{D}W^{D}P$
**CCDC Deposition number**	2465100	2465101
**Chemical formula**	$C_{16}H_{17}N_3O_{2.67}$	$C_{16}H_{17}N_3O_2$
**Crystal system**	Hexagonal	Hexagonal
**Space group**	$P\ 6_3$	$P\ 6_3$
**Mr**	293.99	283.32
**Volume (Å^3)**	2157.73(14)	2094.75(7)
**a(Å)**	15.1442(4)	14.9482(2)
**b (Å)**	15.1442(4)	14.9482(2)
**c (Å)**	10.8636(4)	10.8249(2)
**α (°)**	90	90
**β (°)**	90	90
**γ (°)**	120	120
**Z**	6	6
**Mu (mm^{-1})**	0.095	0.091
**Temperature**	120 K	100.01(10) K
**Data collection Diffractometer**	Rigaku Synergy S	Rigaku Synergy S
	Pilatus 300K (Mo)	Pilatus 300K (Mo)
**No. of reflections(unique)**	24180(4181)	44684(6099)
**θ° range**	3.106 to 31.192°	2.725 to 35.636°
**Reflections/restraints/parameters**	4181/1/196	6099/1/194
**R[F2 > 2σ(F2)]**	0.0387	0.0351
**wR(F2)**	0.1044	0.0949
**Goodness-of-fit**	1.040	1.095
**H-atom treatment**	calc, riding	calc, riding

Supplementary Table 3: Data collection and refinement statistics of O-P and c-VP crystals.

**Crystal data**	**O-P**	**c-VP**
**CCDC Deposition number**	2465132	2504998
**Chemical formula**	$C_{13}H_{15}NO_4$	$C_{20}H_{30}N_4O_4$
**Crystal system**	Orthorhombic	Orthorhombic
**Space group**	$P\ 2_12_12_1$	$P\ 2_12_12_1$
**Mr**	249.26	390.48
**Volume (Å^3)**	1216.26	1980.20
**a(Å)**	6.12576(5)	5.6344(2)
**b (Å)**	7.84368(7)	10.2561(2)
**c (Å)**	25.3131(2)	34.2673(9)
**α (°)**	90	90
**β (°)**	90	90
**γ (°)**	90	90
**Z**	4	4
**Mu (mm^{-1})**	0.844	0.092
**Temperature**	100 K	100 K
**Data collection Diffractometer**	Rigaku Synergy S Pilatus 300K (Mo)	Rigaku Synergy S Pilatus 300K (Mo)
**No. of reflections(unique)**	13082(2481)	8388(8393)
**θ° range**	3.492 to 74.869°	5.338 to 71.52
**Reflections/restraints/parameters**	2481/0/165	8393/0/267
**R[F2 > 2σ(F2)]**	0.0220	0.0424
**wR(F2)**	0.0579	0.1170
**Goodness-of-fit**	1.066	1.033
**H-atom treatment**	calc, riding	mixed

Supplementary Table 4: Data collection and refinement statistics of c-^{L}W^{L}P/c-^{D}W^{L}P and c-^{L}W^{L}P/c-^{D}W^{D}P crystals.

**Crystal data**	**c-^{L}W^{L}P/c-^{D}W^{L}P**	**c-^{L}W^{L}P/c-^{D}W^{D}P**
**CCDC Deposition number**	2465117	2465118
**Chemical formula**	$C_{16}H_{17}N_3O_2$	$C_{16}H_{17}N_3O_2$
**Crystal system**	Monoclinic	orthorhombic
**Space group**	$P\ 2_1$	*P* bca
**Mr**	283.32	283.32
**Volume (Å^3)**	1416.31(4)	2709.97(5)
**a(Å)**	12.1935(2)	10.77816(12)
**b (Å)**	6.70628(12)	10.26718(11)
**c (Å)**	17.3998(3)	24.4889(2)
**α (°)**	90	90
**β (°)**	95.4880(16)	90
**γ (°)**	90	90
**Z**	4	8
**Mu (mm^{-1})**	0.728	0.761
**Temperature**	100 K	100 K
**Data collection Diffractometer**	Rigaku Synergy R HyPix-Arc150 (Cu)	Rigaku Synergy R HyPix-Arc150 (Cu)
**No. of reflections(unique)**	22914(5553)	28879(2774)
**θ° range**	3.642 to 74.749°	3.610 to 74.890°
**Reflections/restraints/parameters**	5553/1/380	2976/0/205
**R[F2 > 2σ(F2)]**	0.0392	0.0274
**wR(F2)**	0.1073	0.0737
**Goodness-of-fit**	1.058	1.053
**H-atom treatment**	calc, riding	mixed

Supplementary Table 5: Data collection and refinement statistics of $^{L}W^{D}P$/c-$^{D}W^{L}P$ and c-$^{L}W^{D}P$/c-$^{D}W^{D}P$ crystals.

Crystal data	c-$^{L}W^{D}P$/c-$^{D}W^{L}P$	c-$^{L}W^{D}P$/c-$^{D}W^{D}P$
**CCDC Deposition number**	2465119	2465120
**Chemical formula**	$C_{16}H_{17}N_3O_2$	$C_{32}H_{32}N_6O_4$
**Crystal system**	Monoclinic	Monoclinic
**Space group**	$P\ 2_1/c$	$P\ 2_1$
**Mr**	283.32	283.33
**Volume (Å^3)**	1404.83(2)	1417.17(5)
**a(Å)**	12.15332(10)	12.1909(2)
**b (Å)**	6.45412(5)	6.70802(13)
**c (Å)**	18.01277(15)	17.4095(4)
**α (°)**	90	90
**β (°)**	96.1259(7)	95.4860(16)
**γ (°)**	90	90
**Z**	4	2
**Mu (mm^{-1})**	0.734	0.090
**Temperature**	100 K	100 K
**Data collection Diffractometer**	Rigaku Synergy R	Rigaku Synergy S
	HyPix-Arc150 (Cu)	Pilatus 300K (Mo)
**No. of reflections(unique)**	26877(2858)	36862(9890)
**θ° range**	3.658 to 74.896°	2.755 to 33.720°
**Reflections/restraints/parameters**	2858/0/195	9890/1/379
**R[F2 > 2σ(F2)]**	0.0340	0.0408
**wR(F2)**	0.0846	0.1129
**Goodness-of-fit**	1.029	1.059
**H-atom treatment**	mixed	mixed

Supplementary Table 6: Data collection and refinement statistics of ^{L}W^{L}P/c-^{L}W^{D}P and c-^{D}W^{L}P/c-^{D}W^{D}P crystals.

**Crystal data**	**c-^{L}W^{L}P/c-^{L}W^{D}P**	**c-^{D}W^{L}P/c-^{D}W^{D}P**
**CCDC Deposition number**	2465116	2465121
**Chemical formula**	$C_{16}H_{16.83}N_3O_{2.17}$	$C_{16}H_{17.33}N_3O_{2.17}$
**Crystal system**	Hexagonal	Hexagonal
**Space group**	$P\,6_3$	$P\,6_3$
**Mr**	285.87	286.38
**Volume (Å^3)**	2122.13(3)	2125.26(6)
**a(Å)**	15.0218(1)	15.0335(2)
**b (Å)**	15.0218(1)	15.0335(2)
**c (Å)**	10.8592(1)	10.8583(1)
**α (°)**	90	90
**β (°)**	90	90
**γ (°)**	120	120
**Z**	6	6
**Mu (mm^{-1})**	0.743	0.742
**Temperature**	100 K	100 K
**Data collection Diffractometer**	Rigaku Synergy R HyPix-Arc150 (Cu)	Rigaku Synergy R HyPix-Arc150 (Cu)
**No. of reflections(unique)**	35591(2912)	35757(2917)
**θ° range**	3.397 to 74.692°	3.395 to 74.546°
**Reflections/restraints/parameters**	2912/2/216	2917/1/209
**R[F2 > 2σ(F2)]**	0.0311	0.0354
**wR(F2)**	0.0781	0.0923
**Goodness-of-fit**	1.087	1.091
**H-atom treatment**	mixed	calc, riding

Supplementary Table 7: Details of the MD simulations

Cyclo-dipeptide		Number of c-WP molecules	Number of crystal water molecules	Number of atoms	Simulation box parameters (a, b, c) (nm) (α, β, γ) (degree)
S-twist	c-^{L}W^{L}P	1296	0	49248	(8.97, 8.97, 6.49) (90, 90, 120)
	c-^{L}W^{D}P	1296	864	51840	(9.09, 9.09, 6.52) (90, 90, 120)
Z-twist	c-^{D}W^{L}P	1296	864	51840	(9.09, 9.09, 6.52) (90, 90, 120)
	c-^{D}W^{D}P	1296	0	49248	(8.97, 8.97, 6.49) (90, 90, 120)

Supplementary Table 8: The number of categorized contacts per unit cell in the triple helical structures.

	Contact number	W main chain	W side chain	P main chain	P side chain
c-^{L}W^{L}P	W main chain	35.9 ± 0.1	23.3 ± 0.1	12.1 ± 0.1	8.4 ± 0.1
	W side chain	23.3 ± 0.1	48.1 ± 0.1	20.0 ± 0.1	33.7 ± 0.1
	P main chain	12.1 ± 0.1	20.0 ± 0.1	11.5 ± 0.1	12.0 ± 0.1
	P side chain	8.4 ± 0.1	33.7 ± 0.1	12.1 ± 0.1	12.0 ± 0.1
c-^{L}W^{D}P	W main chain	32.8 ± 0.2	20.6 ± 0.1	11.0 ± 0.1	11.0 ± 0.1
	W side chain	20.6 ± 0.1	45.6 ± 0.3	17.8 ± 0.1	26.9 ± 0.2
	P main chain	11.0 ± 0.1	17.8 ± 0.1	11.4 ± 0.1	15.8 ± 0.1
	P side chain	10.0 ± 0.1	26.9 ± 0.2	15.8 ± 0.1	12.1 ± 0.1
c-^{D}W^{L}P	W main chain	32.8 ± 0.2	20.6 ± 0.1	11.0 ± 0.1	10.0± 0.1
	W side chain	20.6 ± 0.1	45.6 ± 0.3	17.8 ± 0.1	26.9 ± 0.2
	P main chain	11.0 ± 0.1	17.8 ± 0.1	11.4 ± 0.1	15.8 ± 0.1
	P side chain	10.0 ± 0.1	26.9 ± 0.2	15.8 ± 0.1	12.0 ± 0.1
c-^{D}W^{D}P	W main chain	35.9 ± 0.1	23.3 ± 0.1	12.1 ± 0.1	8.4 ± 0.1
	W side chain	23.3 ± 0.1	48.1 ± 0.1	20.0 ± 0.1	33.7 ± 0.1
	P main chain	12.1 ± 0.1	20.0 ± 0.1	11.5 ± 0.1	12.1 ± 0.1
	P side chain	8.4 ± 0.1	33.7 ± 0.1	12.1 ± 0.1	12.1 ± 0.1

CheckCIF B-level Alerts and Justification

Two B-level alerts are present in the CheckCIF report for c-$^{D}W^{L}P$ (CCDC 2465100). The two B-level alerts arise from isolated oxygen atoms corresponding to structural water molecules. The hydrogen atoms of these water molecules could not be located reliably and were therefore not included in the refinement.

Two B-level alerts are present in the CheckCIF report for c-$^{D}W^{D}P$ (CCDC 2465101). he checkCIF report for c-$^{D}W^{D}P$ shows two B-level alerts (PLAT094 and PLAT097) related to the ratio of maximum/minimum residual density (6.07) and a relatively large positive residual density (1.54 e Å^{-3}). This arises from a residual peak that could not be reasonably modeled in the refinement.

Supplementary references:

1 Langan, P., Mason, S. A., Myles, D. & Schoenborn, B. P. Structural characterization of crystals of α-glycine during anomalous electrical behaviour. *J. Appl. Crystallogr.* **58**, 728-733 (2002).

2 Kolesov, B. A., Minkov, V. S., Boldyreva, E. V. & Drebushchak, T. N. Phase transitions in the crystals of L- and DL-cysteine on cooling: intermolecular hydrogen-bond distortions and side-chain motions of thiol groups. 1. L-cysteine. *J. Phys. Chem. B* **112**, 12827-12839 (2008).

3 Karothu, D. P. et al. Mechanically robust amino acid crystals as fiber-optic transducers and wide bandpass filters for optical communication in the near-infrared. *Nat. Commun.***12**, 1326 (2021).
