# Supplementary material for: Formation of S- and Z-twist supramolecular micro-ropes by peptide stereoisomers
Source: Nat Commun. 2026 Mar 26;17:4424. doi: 10.1038/s41467-026-71043-5 (PMC13183945; doi:10.1038/s41467-026-71043-5)
Supplement: Supplementary file 2 — Description of Additional Supplementary Files [file 41467_2026_71043_MOESM2_ESM.pdf]

Description of Additional Supplementary Files

Supplementary Video 1.

The self-assembly pathway of the c-$^{L}W^{L}P$ peptide. Time-lapse images were captured at one-minute intervals throughout the growth process, with a total duration of 151 minutes.

Supplementary Video 2.

The self-assembly pathway of the c-$^{L}W^{D}P$ peptide. Time-lapse images were captured at one-minute intervals throughout the growth process, with a total duration of 105 minutes.

Supplementary Video 3.

The self-assembly pathway of the c-$^{D}W^{L}P$ peptide. Time-lapse images were captured at one-minute intervals throughout the growth process, with a total duration of 151 minutes.

Supplementary Video 4.

The self-assembly pathway of the c-$^{D}W^{D}P$ peptide. Time-lapse images were captured at one-minute intervals throughout the growth process, with a total duration of 151 minutes.

Supplementary Video 5.

The crystal structure of c-$^{L}W^{L}P$ assemblies showing S-micro-rope-like twists.

Supplementary Video 6.

The crystal structure of c-$^{L}W^{D}P$ assemblies showing S-micro-rope-like twists.

Supplementary Video 7.

The crystal structure of c-^{D}W^{L}P assemblies showing Z-micro-rope-like twists.

Supplementary Video 8.

The crystal structure of c-^{D}W^{D}P assemblies showing Z-micro-rope-like twists.

Supplementary Video 9.

The crystal structure of c-^{L}W^{L}P/c-^{L}W^{D}P co-assemblies showing S-micro-rope-like twists.

Supplementary Video 10.

The crystal structure of c-^{D}W^{L}P/c-^{D}W^{D}P co-assemblies showing Z-micro-rope-like twists.

Supplementary Data 1.

Simulation position of c-^{L}W^{L}P, c-^{L}W^{D}P, c-^{D}W^{L}P, c-^{D}W^{D}P, and O-P crystals.
